# The age factor in optic nerve regeneration: Intrinsic and extrinsic barriers hinder successful recovery in the short‐living killifish

**DOI:** 10.1111/acel.13537

**Published:** 2021-12-19

**Authors:** Sophie Vanhunsel, Steven Bergmans, An Beckers, Isabelle Etienne, Tine Van Bergen, Lies De Groef, Lieve Moons

**Affiliations:** ^1^ Neural Circuit Development and Regeneration Research Group Animal Physiology and Neurobiology Section Department of Biology KU Leuven Leuven Belgium; ^2^ Oxurion NV Heverlee Belgium; ^3^ Leuven Brain Institute Leuven Belgium

**Keywords:** African turquoise killifish, aging, axonal regeneration, central nervous system, optic nerve crush, short lifespan

## Abstract

As the mammalian central nervous system matures, its regenerative ability decreases, leading to incomplete or non‐recovery from the neurodegenerative diseases and central nervous system insults that we are increasingly facing in our aging world population. Current neuroregenerative research is largely directed toward identifying the molecular and cellular players that underlie central nervous system repair, yet it repeatedly ignores the aging context in which many of these diseases appear. Using an optic nerve crush model in a novel biogerontology model, *that is*, the short‐living African turquoise killifish, the impact of aging on injury‐induced optic nerve repair was investigated. This work reveals an age‐related decline in axonal regeneration in female killifish, with different phases of the repair process being affected depending on the age. Interestingly, as in mammals, both a reduced intrinsic growth potential and a non‐supportive cellular environment seem to lie at the basis of this impairment. Overall, we introduce the killifish visual system and its age‐dependent regenerative ability as a model to identify new targets for neurorepair in non‐regenerating individuals, thereby also considering the effects of aging on neurorepair.

## Graphical abstract

In contrast to findings in zebrafish, this study reveals a striking age‐related decline in optic nerve regeneration in the short‐living killifish, with different phases of the repair process being affected depending on the age. Interestingly, while resembling zebrafish at young age, the regenerative ability is more mammalian‐like at old age. As in mammals, both a reduced intrinsic growth potential and a non‐supportive cellular environment seem to lie at the basis of impaired nerve regeneration.

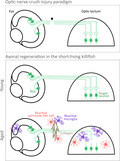

## 1 INTRODUCTION

The fast‐aging African turquoise killifish (*Nothobranchius furzeri*) has arisen as a novel vertebrate biogerontology model as it recapitulates many traits of human aging (Reuter et al., 2018). Despite a broad characterization of aging hallmarks in the killifish, its potential to regrow central nervous system (CNS) axons, and in particular how this capacity alters with age, remains largely unexplored. As a teleost fish, the killifish has an astonishing regenerative ability in its adult CNS, with a high *de novo* neurogenic ability and capability to regrow axons, rebuild circuits, and restore function after injury (Fleisch et al., 2011; Rasmussen & Sagasti, 2017; Zupanc & Sîrbulescu, 2012). However, aging has been shown to affect nervous system functionality, vulnerability to trauma and disease, as well as its regenerative potential, both in non‐vertebrates and vertebrates (Vanhunsel et al., 2020). Identifying the molecules and signaling pathways contributing to this flawed regeneration in an aged setting is therefore of great interest in neuroregenerative research. We propose the killifish CNS, and its age‐dependent repair capacity, as a promising model system to study the mechanisms that underlie functional neural circuit restoration, thereby contributing to new insights on how to stimulate regeneration in the (old) mammalian CNS.

The visual projection is a well‐studied model system to investigate axonal regeneration in the CNS, in mammals as well as fish. The retinofugal projection, bringing visual information from the retina to the brain, consists of the axons of a single retinal cell type, namely the retinal ganglion cells (RGCs), which bundle together to form the optic nerve. In teleosts, RGC axons completely cross over at the optic chiasm and predominately innervate the contralateral optic tectum of the fish brain. The optic tectum of adult fish is considered homologous to the superior colliculus in mammals and is a multilayered structure that can be divided in three main zones: the superficial and central zones, where tectal afferents terminate, and the periventricular gray zone (PGZ), containing the majority of the tectal neuronal cell bodies (Bally‐Cuif & Vernier, 2010; Neuhauss, 2010; Nevin et al., 2010). Restoration of this optic projection after optic nerve injury is an example of the remarkable intrinsic ability of teleost fish to repair axonal damage in the adult CNS. Indeed, upon crush or even transection of the optic nerve, RGCs of adult fish are capable of regrowing their axons, which then navigate to and reinnervate their target cells in the optic tectum to finally restore vision, proving that the visual system of teleosts forms a perfect model system for investigating the different aspects of axonal regrowth (Bollaerts et al., 2018). According to zebrafish and goldfish literature, the repair process of optic nerve regeneration can be subdivided into five phases: (1) the injury response period, occurring at <1 day *post*injury (dpi), which is characterized by shrinkage of RGC dendrites; (2) the axonal outgrowth initiation phase, wherein distal axons of the injured RGCs degenerate and new RGC axons prepare for neurite sprouting, marked by the maximal upregulation of growth‐associated genes in the RGCs; (3) the axon elongation and target reinnervation period, in which RGC axons extend toward their target cells in the brain; (4) the synaptogenesis phase, wherein synapses are repaired and retinotopy is restored, and in which several vision‐dependent reflexes (optokinetic response, dorsal light reflex, and optomotor response) are restored; and (5) a final period involving long‐term synaptic rearrangements and recovery of complex behaviors (Becker & Becker, 2014; Beckers et al., 2019; Bhumika et al., 2015; Diekmann et al., 2015; Kato et al., 1999, 2013; Lemmens et al., 2016; McCurley & Callard, 2010; Van houcke et al., 2017; Zou et al., 2013).

Adopting the optic nerve crush (ONC) model in female killifish, we aim to further unravel the impact of aging on axonal regeneration in the visual system. In this study, after a detailed characterization of the model in young adult fish, we mapped the different phases of the optic nerve repair process in three additional age groups: middle‐aged, old, and very old fish. Depending on the age, repair of axonal damage seems to be affected in different phases of the regenerative process. While middle‐aged fish show a delay in optic nerve regeneration and only partially recover their vision following ONC, old and very old fish do not restore vision at all. Indeed, our data reveal a striking impairment in RGC axonal regrowth in older fish, starting with a delay in axon outgrowth due to a low intrinsic ability to grow axons, and next failing in the subsequent phases of target neuron reinnervation and synaptogenesis. Besides the poor intrinsic growth capacity of older RGCs, we suggest that also a non‐supportive extrinsic environment, actuated by abnormally reactivated micro‐ and macroglial cells, contributes to the observed age‐related loss of functional repair in female killifish. Our results shed light on the underlying aging processes affecting the regenerative potential and thereby contribute to the search for effective neuroregenerative therapies in the (aged) mammalian CNS.

## 2 RESULTS

### 2.1 Detailed characterization of the axonal regeneration process in young adult killifish subjected to ONC

**FIGURE 1:**
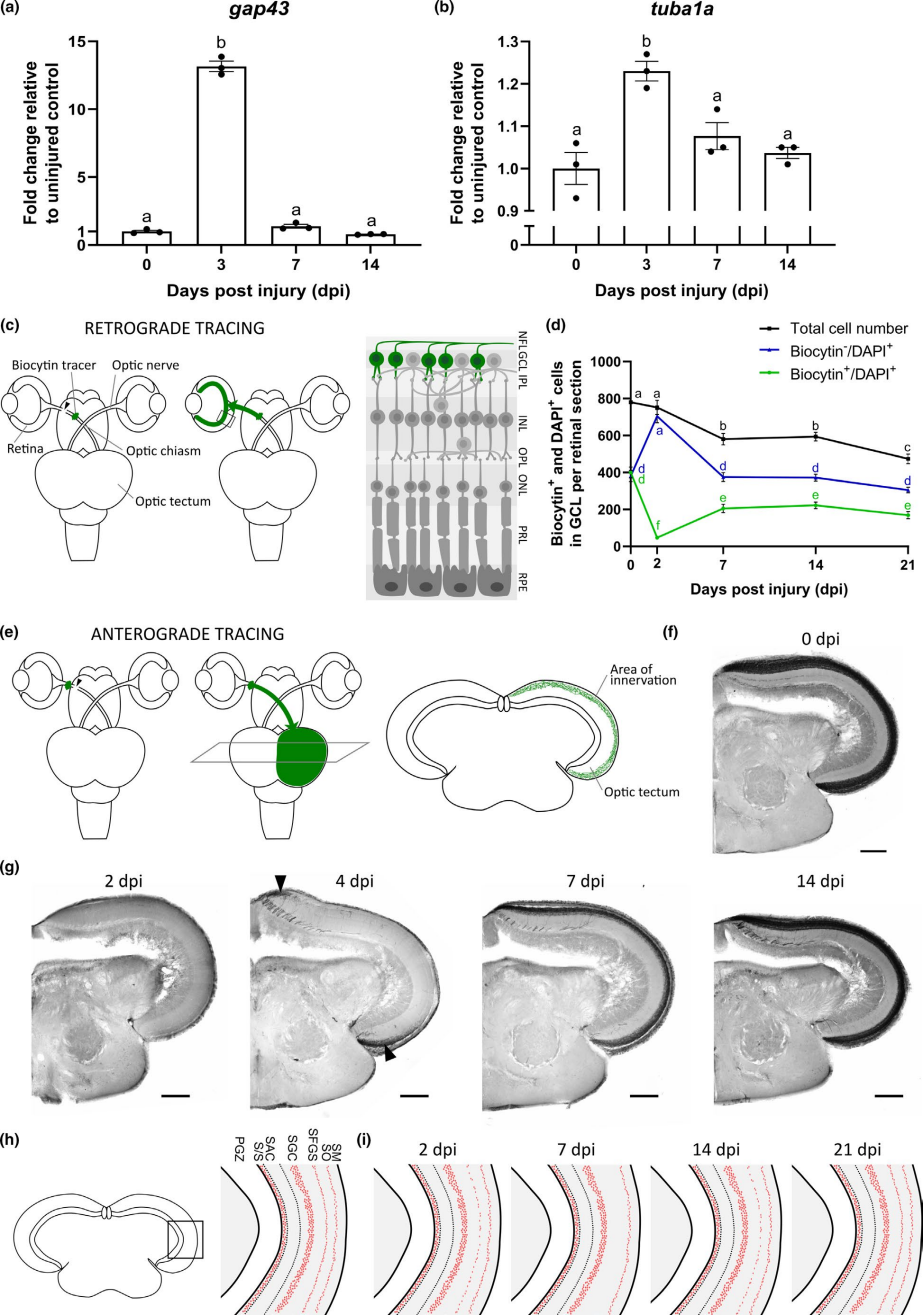
Time course of optic nerve regeneration following optic nerve crush injury in young adult killifish. (a, b) RT‐qPCR for *gap43* (a) and *tuba1a* (b) demonstrates a significant increase in expression levels in the retina at 3 dpi. *n* = 3. (c) Illustration of the retrograde biocytin tracing method, explained in detail in the Experimental Procedures section. (d) Counting the number of DAPI^+^ cells in the GCL upon ONC reveals a pronounced cellular loss between 2 and 7 dpi (black line). The number of biocytin^−^/DAPI^+^ cells is comparable in injured and control retinas, except at 2 dpi when this cell population not only contains displaced amacrine cells but also RGCs of which the axons have not reached the site of tracer placement yet (blue line). The number of biocytin^+^/DAPI^+^ cells rises during the course of regeneration, to reach a plateau at 7 dpi (green line). *n* = 7–10. (e) Illustration of the anterograde biocytin tracing method, explained in detail in the Experimental Procedures section. (f) Microscopic image of a biocytin‐labeled coronal brain section of an uncrushed control fish, revealing black RGC axons innervating the tectum. Scale bar = 200 µm. (g) Representative images depicting tectal reinnervation at several time points following ONC. While no biocytin‐labeled axons are detected in the tectum at 2 dpi, regenerating RGC axons start entering the ventral and dorsal parts of the optic tectum around 4 dpi (arrowheads). From 14 dpi onwards, the biocytin labeling matches that of uninjured fish. Scale bar = 200 µm. (h) Schematic illustration of the synaptic layers in the optic tectum of uncrushed control fish after immunolabeling for synaptotagmin reveals the presence of pre‐synaptic vesicles (red dots) in the SO, SFGS, SGC, and S/S layers of the killifish optic tectum. (i) Following ONC, labeling of pre‐synaptic vesicles starts to decrease in the SFGS layer and is almost completely absent at 7 dpi. At 14 dpi, synaptic labeling increases again, and by 21 dpi, synaptotagmin signals in the SFGS are comparable to that of uninjured fish. All data show mean ± SEM, means with a different letter are significantly different (One‐way ANOVA for panels a and b, Two‐Way ANOVA for panel d), see Table S1 for exact *p*‐values. DAPI, 4′,6‐diamidino‐2‐phenylindole; dpi, days *post*injury; *gap43*, *growth*‐*associated protein 43*; GCL, ganglion cell layer; INL, inner nuclear layer; IPL, inner plexiform layer; NFL, nerve fiber layer; ONC, optic nerve crush; ONL, outer nuclear layer; OPL, outer plexiform layer; PGZ, periventricular gray zone; PRL, photoreceptor segment layer; RGC, retinal ganglion cell; RPE, retinal pigment epithelium; RT‐qPCR, real‐time quantitative polymerase chain reaction; S/S, projection zone between SAC and PGZ; SAC, *stratum album centrale*; SFGS, *stratum fibrosum et griseum superficiale*; SGC, *stratum griseum centrale*; SM, *stratum marginale*; SO, *stratum opticum*; *tuba1a*, *tubulin alpha 1a chain*

To characterize optic nerve regrowth in the young adult female killifish, we evaluated the different regenerative phases following ONC in 6‐weeks‐old young adult fish. We first assessed the initiation of axonal regrowth by determining retinal mRNA levels of *growth*‐*associated protein 43* (*gap43*) and *tubulin alpha 1a chain* (*tuba1a*), both validated markers for the intrinsic axonal outgrowth potential of RGCs (Beckers et al., 2019; Kato et al., 2013; McCurley & Callard, 2010; Van houcke et al., 2017), at 0, 3, 7, and 14 dpi. *Gap43* (Figure 1a) and *tuba1a* (Figure 1b) mRNA levels were significantly upregulated at 3 dpi, followed by a decline in expression to baseline values by 7 dpi. Killifish RGCs thus transiently express these growth‐associated markers to initiate axonal regrowth (Table 1).

**TABLE 1:** Overview of the phases of the regenerative process following optic nerve injury in three different fish models of young adult age, *that is*, *Danio rerio*, *Carassius auratus*, *and Nothobranchius furzeri*

Phases of the regenerative process following optic nerve injury	*D. rerio*	*C. auratus*	*N. furzeri*
Injury response	<1 dpi		
Outgrowth initiation	1–7 dpi	1–5 dpi	<7 dpi
Synthesis RNA/proteins for neurite sprouting	1–7 dpi		<7 dpi
Number of regenerating RGCs in retina starts increasing	2 dpi		2 dpi
Axon elongation and target reinnervation	5–30 dpi	14–40 dpi	4–14 dpi
Axons appear in the optic tectum	5–7 dpi	14–20 dpi	4 dpi
Complete reinnervation of the optic tectum	10–30 dpi	30–40 dpi	14 dpi
Synaptic repair and restoration retinotopy	14–80 dpi		
Complete reformation of synapses / OKR	14 dpi		14–21 dpi
DLR	18 dpi		21 dpi
Re‐establishment retinotopy / OMR	25–63 dpi		
Long‐term synaptic rearrangements and recovery complex behaviors	60–120 dpi	150–180 dpi	
Chasing and/or shoaling behavior	60–100 dpi	150–180 dpi	
References	McCurley and Callard (2010) Kato et al. (2013) Zou et al. (2013) Becker and Becker (2014) Bhumika et al. (2015) Diekmann et al. (2015) Lemmens et al. (2016) Van houcke et al. (2017) Beckers et al. (2019)	Kato et al. (1999) Kato et al. (2013)	Current study

Note

Given values are approximate.

Abbreviations: DLR, dorsal light reflex; dpi, days *post*injury; OKR, optokinetic response; OMR, optomotor response; ONI, optic nerve injury; RGC, retinal ganglion cell

Outgrowth initiation was further investigated using retrograde biocytin tracing in 6‐weeks‐old fish, followed by analysis on midsagittal retinal sections (Figure 1c). In baseline conditions, this retrograde tracing results in labeling of all RGCs in the retinal ganglion cell layer (GCL). Analyzing the proportion of biocytin^+^/4′,6‐diamidino‐2‐phenylindole (DAPI)^+^ and biocytin^−^/DAPI^+^ cells revealed that, similar as reported in other fish species (Mack et al., 2004), 51.2 ± 3.7% of the cells in the GCL are RGCs that take up the dye (biocytin^+^/DAPI^+^) and 48.8 ± 3.9% of the cells are displaced amacrine cells (biocytin^−^/DAPI^+^) (Figure 1d). Upon ONC, however, cell numbers and cell type proportions changed. Counting the total number of DAPI^+^ cells in the GCL of retinal midsagittal sections demonstrated a pronounced cell loss between 2 and 7 dpi that resulted in a 39.3 ± 3.4% reduction in total cell number by 21 dpi (Figure 1d). To confirm the occurrence of cell death following ONC, the number of activated‐caspase‐3^+^ cells in the GCL was quantified. The number of apoptotic cells tended to increase in the injured retinas when compared to uninjured ones, with a maximum number at 7 dpi that decreased again by 14 dpi (0.3 ± 0.2 apoptotic cells in the GCL per midsagittal retinal section in uninjured fish, 3.1 ± 1.0 cells at 7 dpi, 0.9 ± 0.1 cells at 14 dpi), indicating that apoptosis indeed occurs in young adult killifish subjected to ONC (see also Figure 3c). Next, to disentangle whether the observed cell loss in the GCL resulted from RGC loss only, or from loss of both RGCs and displaced amacrine cells, the proportion of biocytin^−^/DAPI^+^ and biocytin^+^/DAPI^+^ cells was evaluated in retinas of nerve crushed fish. At 2 dpi, the number of biocytin^−^/DAPI^+^ cells exceeded that of uninjured fish as this cell population not only contained displaced amacrine cells, but also RGCs of which the axons did not reach the site of tracer placement yet. At all later time points *post*‐ONC, the number of biocytin^−^/DAPI^+^ cells remained constant and comparable to baseline values, indicating that the ~40% loss of cells in the GCL is not attributable to loss of displaced amacrine cells (Figure 1d). The number of biocytin^+^/DAPI^+^ RGCs, however, showed a permanent decrease as a result of ONC. First, at 2 dpi, their number was significantly reduced compared to uninjured numbers as not all RGC axons reached the site of tracer placement yet (with 11.8 ± 1.2% of the RGCs, relative to those traced in the uninjured retina, arriving at the site of biocytin tracer placement). The number of biocytin^+^/DAPI^+^ RGCs then increased and reached a plateau at 7 dpi. However, with 51.4 ± 5.6% of the total number of RGCs labeled at 7 dpi, it never reapproached uninjured levels over the course of regeneration (Figure 1d). Strikingly, these data indicate that, of the total RGC population, ~40% is lost following ONC, ~50% is able to regenerate, and the remaining ~10% seems to stay alive in the retina without regrowing an axon.

We next evaluated the timelines of the axon elongation and tectal reinnervation phase. Target reinnervation following ONC was studied via anterograde biocytin tracing, followed by analysis of axonal density levels within the RGC axon innervation area on midcoronal optic tectum sections (Figure 1e). In uninjured control fish, RGC axons could be visualized in the *stratum fibrosum et griseum superficiale* (SFGS) and *stratum opticum* (SO) of the tectum (Figure 1f). No biocytin‐labeled RGC axons were detected at 2 dpi, showing that they did not reach the tectum yet. Axons started re‐entering the dorsal and ventral parts of the optic tectum by 4 dpi. At 7 dpi, reinnervation of the tectal layers was still ongoing and analysis of axonal density levels revealed that 90.6 ± 2.0% of the tectal reinnervation area was reinnervated, as compared to uninjured fish. Reinnervation was completed at 14 dpi, with 98.49 ± 0.9082% of the tectal area reinnervated (Figure 1g). In young adult females subjected to ONC, it thus takes about 14 days for the optic tectum to be completely reinnervated by RGC axons (Table 1).

In a following step, the synaptogenesis phase was studied using immunostainings for synaptotagmin (Znp‐1 antibody) (Beckers et al., 2019; Fox & Sanes, 2007; Zappa Villar et al., 2021), a calcium sensor protein present in the membrane of pre‐synaptic vesicles, on optic tectum sections at several time points after ONC. Pre‐synaptic vesicles were clearly visible in the SO, SFGS, *stratum griseum centrale* (SGC), and S/S (projection zone between the *stratum album centrale* (SAC) and PGZ) tectal layers of uninjured fish (Figure 1h), but disappeared following ONC, particularly in the SFGS, which is the tectal layer to which most RGC axons project in teleost fish (Bally‐Cuif & Vernier, 2010; Neuhauss, 2010; Nevin et al., 2010). This degradation of synapses started from 2 dpi onwards in the young adult killifish and continued up to 7 dpi, at which almost no synapses could be observed in the SFGS. At 14 dpi, we noted an injury‐induced synaptogenesis, and synaptic numbers approached pre‐injury levels by 21 dpi (Figure 1i). Thus, injury‐induced synaptogenesis in 6‐weeks‐old killifish occurs in the second and third week *post*‐ONC (Table 1).

**FIGURE 2:**
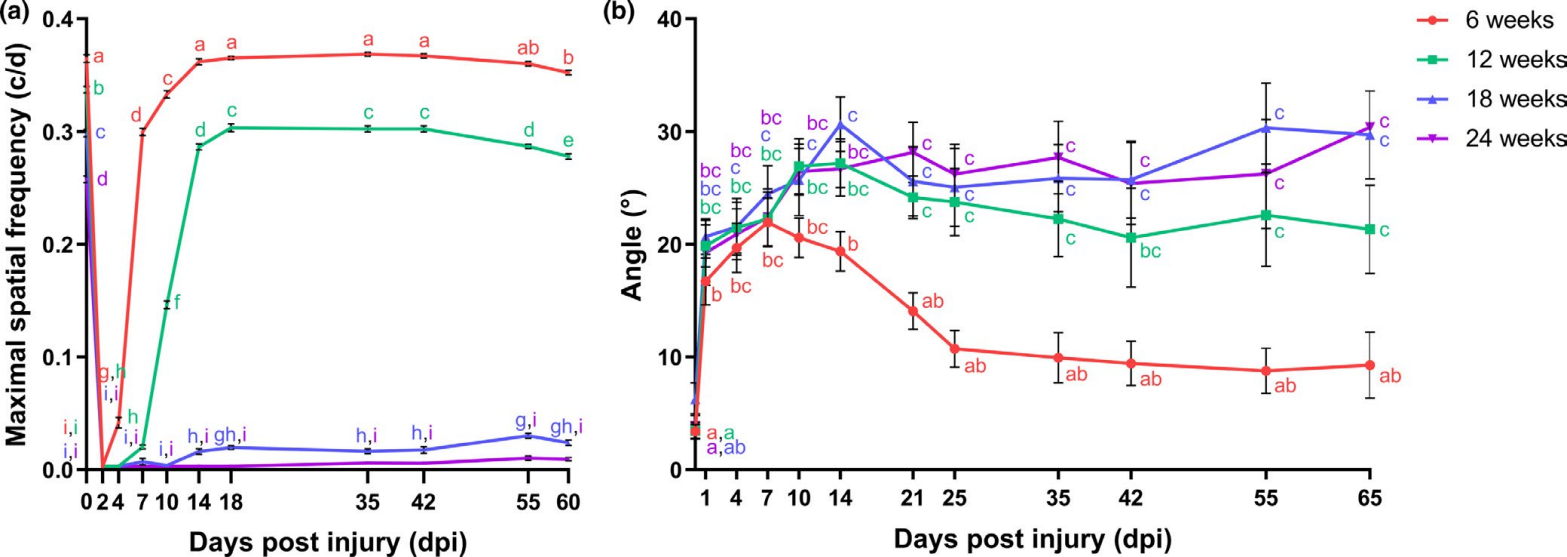
Recovery of primary vision following optic nerve crush injury is impaired in aged killifish. (a) Determining the maximal spatial frequency provoking an optokinetic response at different time points *post*bilateral ONC discloses that young adult killifish start reshowing this reflex from 4 dpi onwards and full recovery is achieved by 14 dpi. In 12‐weeks‐old fish, the response returns from 7 dpi on, yet maximal spatial frequency values never approach uninjured values. Both 18‐weeks‐ and 24‐weeks‐old fish do not regain the optokinetic response after ONC. *n* = 6–10, except for 6‐weeks‐old fish at 10 dpi (*n* = 5) and for 24‐ weeks‐old fish at 18 dpi (*n* = 4). (b) Assessing the degree of tilting at several time points after unilateral ONC reveals almost complete restoration of the dorsal light reflex from 21 dpi onwards in 6‐weeks‐old killifish. Fish of the older age groups, *that is*, 12‐weeks‐, 18‐weeks‐, and 24‐weeks‐old fish, never regain this reflex and remain to swim in a tilted position. *n* = 5–9, except for 18‐weeks‐old fish at 0 dpi (*n* = 4), and 24‐weeks‐old fish at 55 dpi (*n* = 3) and 65 dpi (*n* = 1). All data are represented as mean ± SEM, means with a different letter are significantly different (Two‐Way ANOVA), see Table S1 for exact *p*‐values. dpi, days *post*injury; ONC, optic nerve crush

To evaluate functional recovery after ONC, two different behavioral tests were used. First, an optokinetic response test was performed to assess visual acuity of fish subjected to a bilateral ONC. This test revealed that immediately after ONC, eyesight was completely lost. However, over time, the young adult fish regained their primary vision. Visual acuity was retrieved from 4 dpi onwards and reached baseline values by 14 dpi (Figure 2a). Next, we evaluated the dorsal light reflex. Upon unilateral nerve damage, fish have been demonstrated to alter their swimming position and swim oblique toward the side of the uninjured eye. During the course of regeneration, this oblique swimming position gradually returns to baseline levels (Diekmann et al., 2015). Similar as for the optokinetic response, young adult killifish lost primary vision in their left eye directly after ONC, as they started to swim oblique already at 1 dpi when compared to uninjured fish. Tilting of their frontal body axis increased, reaching a maximum at 7 dpi, and then normalized with progressing axonal regeneration. Indeed, starting from 21 dpi, body axis tilting was no longer significantly different when compared to baseline levels (Figure 2b). Altogether, primary vision has thus completely recovered by 21 dpi in young adult female killifish (Table 1).

### 2.2 Impaired functional recovery following ONC in aged killifish

In a next set of experiments, we aimed at evaluating whether older fish recover from optic nerve damage equally well as young adult killifish. Visual recovery was determined using the optokinetic response test in middle‐aged (12 weeks), old (18 weeks), and very old (24 weeks) fish at various time points following ONC (Figure 2a). Middle‐aged fish showed a delay in functional recovery, regaining visual acuity from 7 dpi in comparison with 6‐weeks‐old fish that already displayed the first signs of recovery at 4 dpi. However, and in contrast to young fish, the optokinetic reflex was never restored to uninjured values at the measured time points, suggesting an age‐related impairment in the regeneration process. Strikingly, the older age groups (18 weeks and 24 weeks) never showed any recovery of this primary reflex, not even after more than 8 weeks, indicating that these older fish did not succeed in restoring the retinotectal circuit after ONC.

We also determined the dorsal light reflex in 12‐weeks‐, 18‐weeks‐, and 24‐weeks‐old fish (Figure 2b). Notably, normalization of body axis tilting could not be detected in any of these age groups. They reached their maximum recovery between 14 and 21 dpi, but, even after 65 days, remained to swim in a tilted position when compared to uninjured controls of the same age group. Middle‐aged, old, and very old fish thus did not regain this reflex following ONC. All in all, our findings show a clear impairment of functional recovery following optic nerve damage in female killifish of older age.

### 2.3 Age‐associated alterations in the intrinsic growth potential of RGC axons after ONC

**FIGURE 3:**
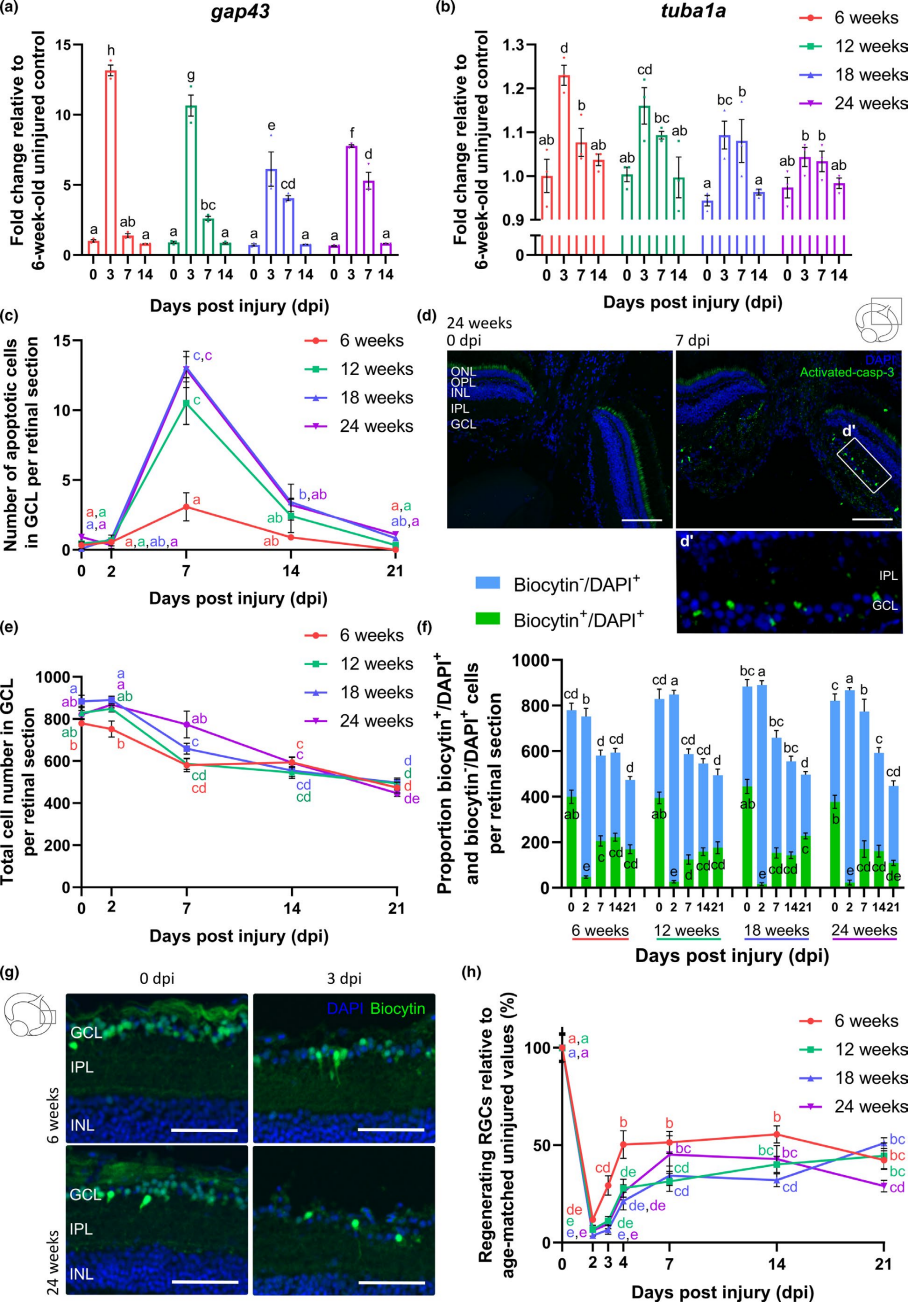
Aging impacts optic nerve regeneration already early in the regenerative process. (a, b) mRNA expression studies reveal reduced *gap43* (a) and *tuba1a* (b) levels at 3 dpi, respectively, in all older age groups and in 18‐weeks‐ and 24‐weeks‐old killifish, as compared to young adult killifish. *n* = 3. (c) Quantification of activated‐caspase‐3 immunopositive apoptotic cells in the GCL of fish subjected to ONC discloses that significantly more cells undergo cell death at 7 dpi in the older age groups when compared to 6‐weeks‐old fish. *n* = 3–5. (d) Representative images of retinal sections of 24‐weeks‐old fish after immunostaining for activated‐caspase‐3 show an increased number of apoptotic cells in the GCL at 7 dpi, predominantly in the central retina. Scale bar = 100 µm. Boxed area is magnified in (d’). (e) Following ONC, the total cell number (DAPI^+^) in the GCL decreases in all older age groups. *n* = 6–10. (f) Analysis of the proportion of biocytin^−^/DAPI^+^ and biocytin^+^/DAPI^+^ cells in the GCL using retrograde biocytin tracing reveals that, while the number of biocytin^−^/DAPI^+^ cells remains rather constant, the number of biocytin^+^/DAPI^+^ cells fails to reach uninjured levels during the regenerative process. *n* = 6–10. (g) Representative images of the retrograde biocytin experiment depict a lower number of RGCs outgrowing their axons past the crush site (biocytin^+^/DAPI^+^) in 24‐weeks‐old fish from 3 dpi on. Scale bar = 50 µm. (h) Quantification of the percentage of biocytin^+^/DAPI^+^ RGCs (relative to the total number of biocytin^+^/DAPI^+^ cells in the GCL of uninjured control fish of the same age group) reveals a clear reduction in the number of regenerating RGCs in all older fish during the early phases of the regenerative process. *n* = 6–10. All values are shown as mean ± SEM, statistical difference is indicated using different letters (Two‐way ANOVA), see Table S1 for exact *p*‐values. Note that the data from 6‐weeks‐old fish are identical to those depicted in Figure 1. DAPI, 4′,6‐diamidino‐2‐phenylindole; dpi, days *post*injury; GCL, ganglion cell layer; INL, inner nuclear layer; IPL, inner plexiform layer; ONC, optic nerve crush; ONL, outer nuclear layer; OPL, outer plexiform layer; RGC, retinal ganglion cell

As a diminished intrinsic growth potential of RGCs might underlie the observed age‐related defect in functional recovery, retinal mRNA expression levels of the growth‐associated genes *gap43* and *tuba1a* were investigated in older killifish. Similar to 6‐weeks‐old fish, real‐time quantitative polymerase chain reaction (RT‐qPCR) on retinal tissues demonstrated an upregulation of *gap43* mRNA in 12‐weeks‐, 18‐weeks‐, and 24‐weeks‐old fish following ONC. However, its expression level at 3 dpi was reduced when compared to young adult fish, suggesting a diminished ability to initiate axon regrowth. Notably, as *gap43* mRNA levels were still elevated at 7 dpi, the outgrowth response seems to be delayed as well (Figure 3a). Additionally, *tuba1a* mRNA expression was lower or even absent in older fish subjected to ONC in comparison with 6‐weeks‐old fish (Figure 3b). These data clearly indicate that aging impacts the intrinsic ability of RGCs to initiate axon outgrowth.

### 2.4 Occurrence of apoptosis and reduced number of regenerating RGCs following ONC in older killifish

To investigate whether ONC also results in apoptosis of RGCs in the older age groups, we quantified the number of activated‐caspase‐3^+^ cells in the GCL of 12‐weeks‐, 18‐weeks‐, and 24‐weeks‐old fish and demonstrated that the number of dying cells in the GCL at 7 dpi was significantly higher in comparison with 6‐weeks‐old fish, with an increased number still visible at 14 dpi (Figure 3c). Interestingly, apoptotic cells were mainly found in the central retina of these aged fish, where the older, presumably more vulnerable, cells are located (Figure 3d, magnification in d’).

Cell loss after nerve damage was also studied using retrograde biocytin tracing in 12‐weeks‐, 18‐weeks‐, and 24‐weeks‐old fish at several time points *post*‐ONC. Similar as observed in young adult fish, the total DAPI^+^ number in the GCL declined after ONC, again resulting in a ~40% reduction in total cell number by 21 dpi (39.1 ± 3.2%, 42.9 ± 1.8%, and 45.6 ± 1.8% reduction in total cell number at 21 dpi in 12‐weeks‐, 18‐weeks‐, and 24‐weeks‐old fish, respectively) (Figure 3e). Counting the number of biocytin^−^/DAPI^+^ and biocytin^+^/DAPI^+^ cells revealed that ONC does not result in death of displaced amacrine cells but eliminates RGCs (Figure 3f). Together with the observed increase in the number of apoptotic cells, these results show that ONC induces RGC death in aged females, just like in young adults.

Using this retrograde tracing paradigm, we also looked more rigorously into age‐associated differences in the number of RGCs that are regenerating, and thus whether the decreased intrinsic outgrowth potential of aged RGCs led to a reduced axonal outgrowth. As compared to young adult fish, the number of regenerating RGCs with axons extending up till the site of biocytin placement was visibly reduced at 3 and 4 dpi in 12‐weeks‐, 18‐weeks‐, and 24‐weeks‐old fish, indicating that axonal outgrowth is indeed affected in the early phases of regeneration (Figure 3g,h). By 21 dpi, 12‐weeks‐ and 18‐weeks‐old fish seemed to have caught up with the young adults as they showed a similar number of axon‐regrowing RGCs, suggestive for a delay in axonal regrowth, rather than a defect. This contrasts findings in 24‐weeks‐old killifish, in which the number of RGCs with regenerating axons tended to remain lower (with 29.0 ± 3.0% of the RGCs outgrowing an axon at 21 dpi). While axonal outgrowth thus seems to be decelerated in middle‐aged and old killifish, a stronger hindrance of outgrowth might occur in very old fish (Figure 3h).

### 2.5 Reduced tectal reinnervation in aged killifish after ONC

**FIGURE 4:**
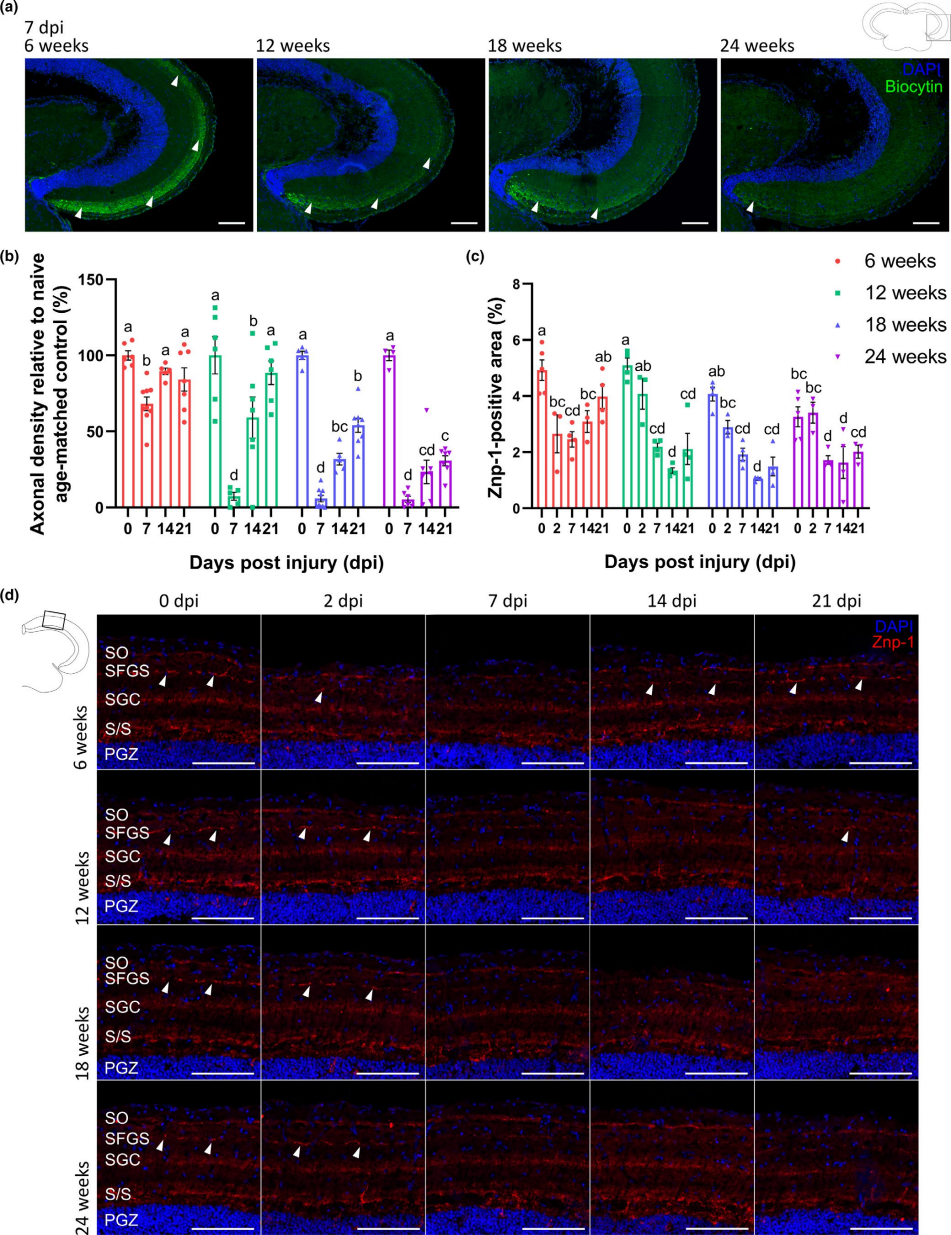
Tectal reinnervation and injury‐induced synaptogenesis are impaired in older killifish. (a) Representative images of coronal optic tectum sections, stained for biocytin after anterograde biocytin tracing, visualize a gradual reduction of RGC axonal density levels (arrowheads) in the tectal reinnervation area at 7 dpi in 12‐weeks‐, 18‐weeks‐, and 24‐weeks‐old fish when compared to 6‐weeks‐old fish. Scale bar = 100 µm. (b) Quantification of axonal density levels, defined as the ratio of the biocytin^+^ area to the total area of RGC innervation in the optic tectum, shows a clear decrease in tectal reinnervation at 7 and 14 dpi in all older age groups. In middle‐aged fish, tectal reinnervation at 21 dpi is comparable to that in uninjured age‐matched controls. Axonal density levels in the optic tectum of 18‐weeks‐ and 24‐weeks‐old fish never approach uninjured values. *n* = 4–9. (c) Quantification of the Znp‐1^+^ area in the SFGS layer of the optic tectum shows a downregulation of the pre‐synaptic signal at 2 dpi in 6‐weeks‐old fish, and at 7 dpi in 12‐weeks‐, 18‐weeks‐, and 24‐weeks‐old fish. While synaptotagmin expression in the SFGS increases again and is comparable to uninjured control levels at 21 dpi in young fish, it does not reapproach baseline levels at any timepoint after ONC in the older fish. *n* = 3–5. (d) Immunostainings for synaptotagmin on coronal brain sections reveal the presence of pre‐synaptic vesicles in the killifish tectum, with synapses in the SFGS layer indicated with arrowheads. Note that the 6‐weeks‐old data are derived from the same data as those illustrated in Figure 1h,i. Scale bar = 100 µm. All data are represented as mean ± SEM, means with a different letter are significantly different (Two‐Way ANOVA), see Table S1 for exact *p*‐values. DAPI, 4′,6‐diamidino‐2‐phenylindole; dpi, days *post*injury; ONC, optic nerve crush; PGZ, periventricular gray zone; RGC, retinal ganglion cell; S/S, projection zone between SAC and PGZ; SAC, *stratum album centrale*; SFGS, *stratum fibrosum et griseum superficiale*; SGC, *stratum griseum centrale*; SO, *stratum opticum*

Via anterograde biocytin tracing and morphometrical quantification on midcoronal tectal sections, we next investigated whether the impaired functional recovery in the older age groups is linked to a diminished RGC axon reinnervation of the optic tectum. At 7 dpi, RGC axonal density levels in the SO and SFGS tectal layers were significantly reduced in 12‐weeks‐, 18‐weeks‐, and 24‐weeks‐old fish as compared to 6‐weeks‐old fish (Figure 4a). Nevertheless, axonal density measurements reapproached uninjured levels at 21 dpi for 12‐weeks‐old fish. The axonal density levels in the optic tectum of 18‐weeks‐ and 24‐weeks‐old fish, on the other hand, did not return to uninjured values at 21 dpi (Figure 4b). Axon elongation thus takes longer or might even be permanently impaired in these older females, eventually resulting in delayed or incomplete reinnervation of the optic tectum.

### 2.6 Impeded synaptic repair in the optic tectum following ONC in aged killifish

To study whether also defects in synaptic repair in the optic tectum lie at the basis of the impaired visual recovery in older fish, synaptic dynamics after ONC were investigated in 12‐weeks‐, 18‐weeks‐, and 24‐weeks‐old fish and compared to those in young adult animals. Immunostainings for synaptotagmin at 0, 2, 7, 14, and 21 dpi showed that degradation of RGC pre‐synaptic vesicles seemed to start later in the older age groups, *that is*, at 7 dpi in fish with 12 weeks, 18 weeks, and 24 weeks of age instead of at 2 dpi in young adult fish. While new synaptic vesicles were already noticeable in the SFGS tectal layer of 6‐weeks‐old fish at 14 dpi, reappearance of pre‐synaptic vesicles only became visible in 12‐weeks‐old fish from 21 dpi onwards. Strikingly, this was not the case in 18‐weeks‐ and 24‐weeks‐old fish, where the reformation of synapses remained non‐existent within the time frame studied (Figure 4c,d). These data indicate a clear effect of aging on injury‐induced synaptogenesis, with a delayed or even absent synaptic repair in middle‐aged and old/very old fish, respectively.

### 2.7 Contribution of an altered inflammatory response and glial reactivity to hindered regeneration

**FIGURE 5:**
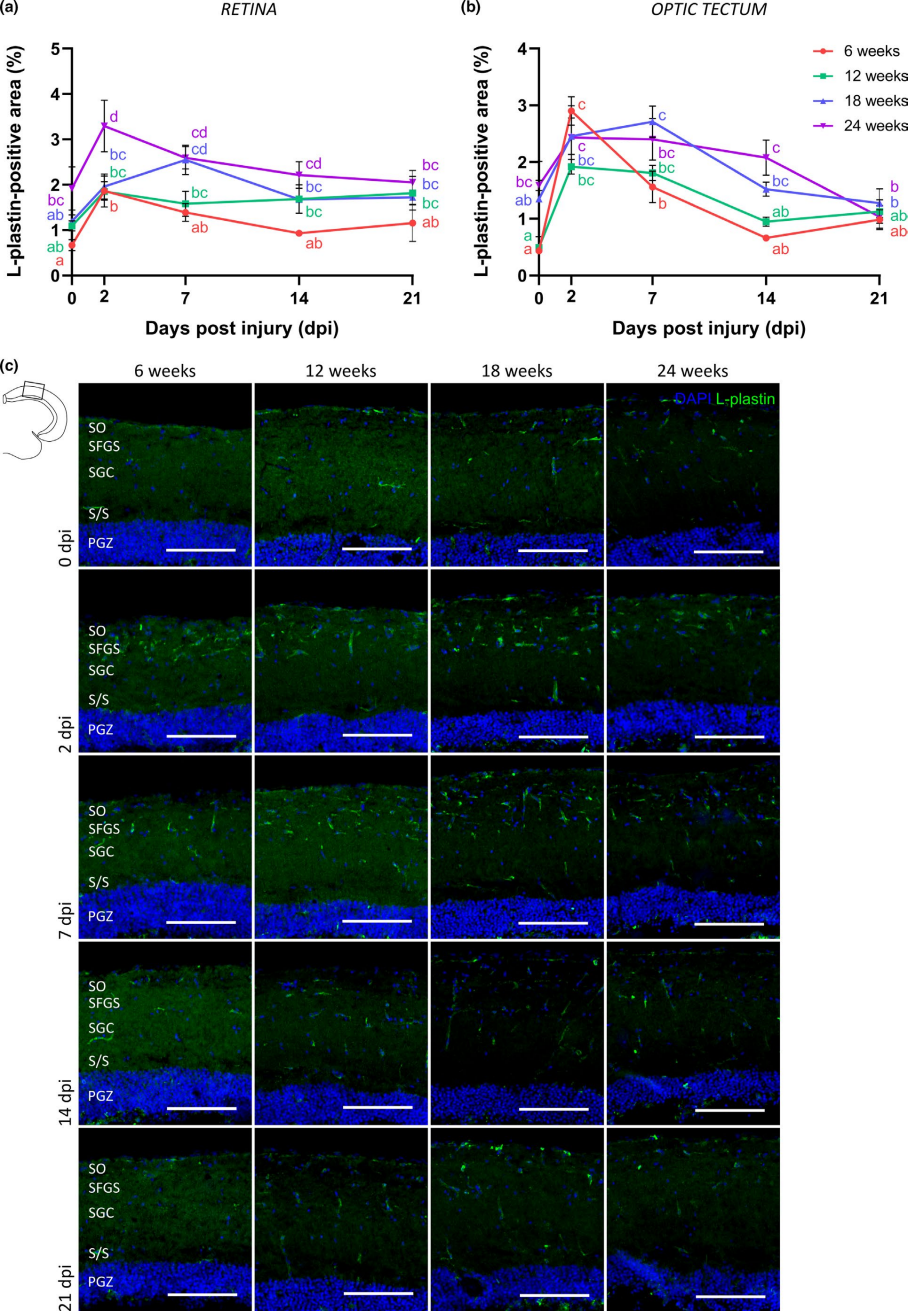
Immune cells respond differently to optic nerve crush injury in aged killifish. (a, b) Quantification of the L‐plastin^+^ area in the retina (a) and optic tectum (b) reveals a transient rise in immune cell number in young adult killifish. The immune response following crush in killifish of older age is more extensive and/or prolonged. *n* = 3–5. (c) Representative pictures of L‐plastin‐stained tectal sections of young adult, middle‐aged, old, and very old killifish reveal an increased number of immune cells in the uninjured aged fish tectum. When subjected to ONC, the immune response seems to be more prolonged in killifish of older age, which is most clear in the tectum of fish at 18 weeks and 24 weeks of age. Scale bar = 100 µm. All values represent mean ± SEM, means with a different letter are significantly different (Two‐Way ANOVA), see Table S1 for exact *p*‐values. DAPI, 4′,6‐diamidino‐2‐phenylindole; dpi, days *post*injury; ONC, optic nerve crush; PGZ, periventricular gray zone; S/S, projection zone between SAC and PGZ; SAC, *stratum album centrale*; SFGS, *stratum fibrosum et griseum superficiale*; SGC, *stratum griseum centrale*; SO, *stratum opticum*

Besides the RGC intrinsic decline in axonal outgrowth, reinnervation, and synaptic restoration potential, we also determined age‐related effects of extrinsic factors that might affect circuit repair following ONC. Previous data from our group disclosed an upregulation of pro‐inflammatory cytokines and an increased presence of resident and/or blood‐born immune cells in the retina and optic tectum of older killifish, indicative of an inflammaging status in the old fish CNS (Vanhunsel et al., 2021). Immunohistochemistry for the pan‐leukocyte marker L‐plastin, and subsequent quantification of the immunopositive area, indeed revealed an increase in microglia/leukocyte number in the retina (Figure 5a, Figure S1) and tectum (Figure 5b,c) of uninjured older killifish as compared to uninjured 6‐weeks‐old animals. Following optic nerve injury, an inflammatory response was induced in both tissues of all age groups. In 6‐weeks‐old fish, immune cell numbers were elevated at 2 dpi and returned to control values around 14 dpi, both in the retina (Figure 5a, Figure S1) and tectum (Figure 5b,c). In the older age groups, and especially in 18‐weeks‐ and 24‐weeks‐old fish, this increase in immune cells lasted longer in the retina as well as the tectum and was more extensive in the retina of 24‐weeks‐old fish as opposed to young adults (Figure 5, Figure S1). Additionally, RT‐qPCR for several pro‐inflammatory cytokines was performed on total retinal and tectal samples. This revealed an overall increased and extended, albeit not always statistically significant, expression of *interleukin* (*il)*‐1β, *tumor necrosis factor* (*tnf*), *il*‐*6*, and *il*‐*8* in the retina and/or tectum of 24‐weeks‐old killifish in comparison with 6‐weeks‐old fish at various time points after injury (Figure S2). Next to increased transcriptional variability in the old age group, the absence of significant findings might be the result of using complete retinal/tectal lysates and thus a dilution effect, as only a small fraction of all cells in the retina and optic tectum is represented by inflammatory cells. All in all, our results do point toward a stronger and/or prolonged immune response in the retina and optic tectum of older fish subjected to ONC, which might affect the circuit repair process.

**FIGURE 6:**
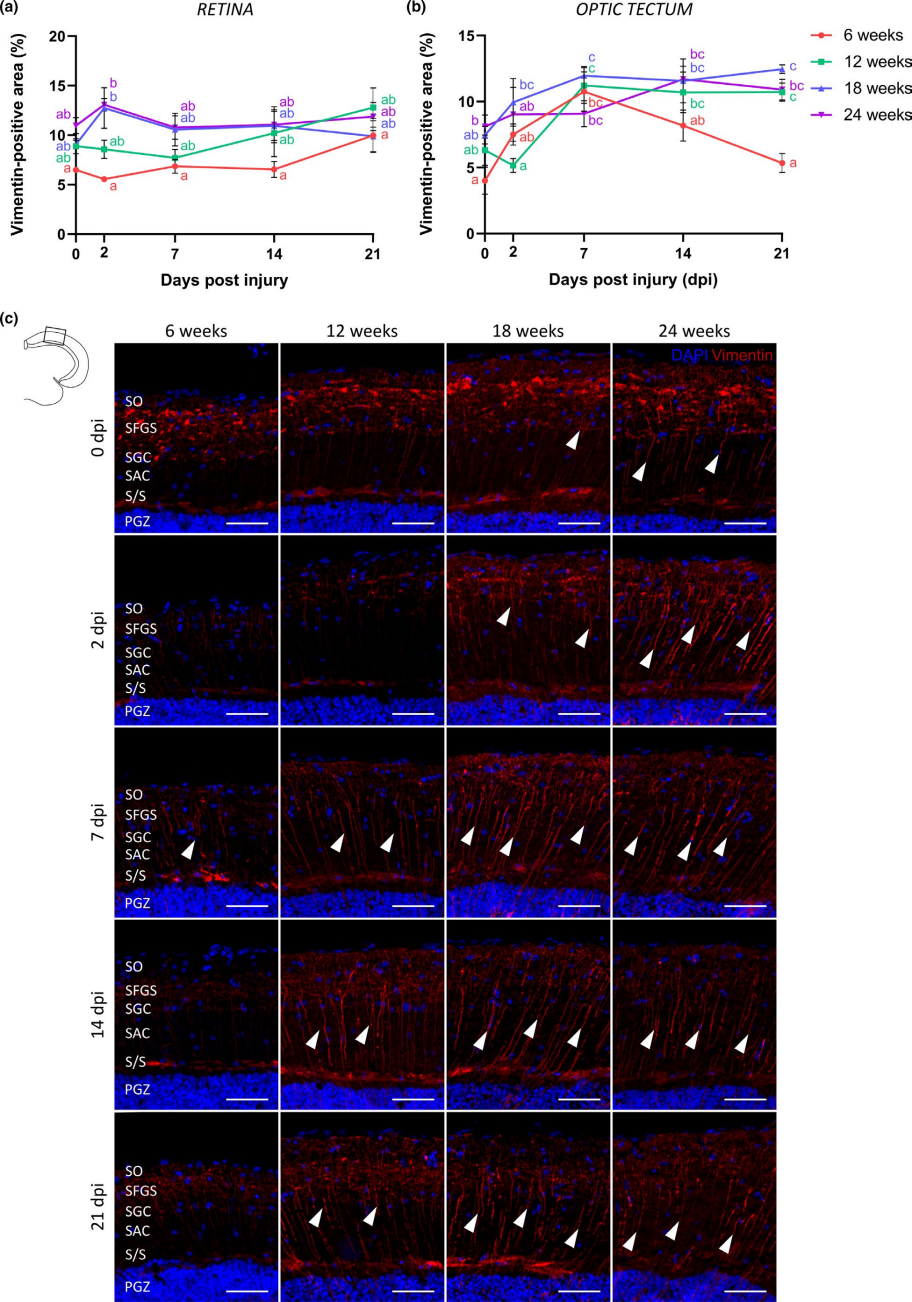
Radial glia in the old killifish optic tectum behave differently in response to optic nerve crush. (a) Quantification of the vimentin^+^ area in the IPL and INL of the retina reveals that ONC does not change the vimentin expression pattern in the retina of fish at any age. *n* = 3–4. (b) Quantification of the vimentin^+^ area in the SGC, SAC, and S/S layers of the optic tectum shows an age‐related upregulation of basal vimentin staining in the glial fibers. Following ONC, an increase in vimentin immunoreactivity is observed in the fibers of young adult killifish, particularly at 7 dpi. Upregulation in the radial fibers *post*‐ONC is more pronounced and prolonged in the older age groups. *n* = 3–4. (c) Representative images showing immunostaining for vimentin in the optic tectum of young adult, middle‐aged, old, and very old killifish, which reveals a gliotic response in the fibers of radial glia (arrowheads) in all age groups subjected to ONC. Scale bar = 50 µm. All values represent mean ± SEM, means with a different letter are significantly different (Two‐Way ANOVA), see Table S1 for exact *p*‐values. DAPI, 4′,6‐diamidino‐2‐phenylindole; dpi, days *post*injury; INL, inner nuclear layer; IPL, inner plexiform layer; ONC, optic nerve crush; PGZ, periventricular gray zone; S/S, projection zone between SAC and PGZ; SAC, *stratum album centrale*; SFGS, *stratum fibrosum et griseum superficiale*; SGC, *stratum griseum centrale*; SO, *stratum opticum*

Additionally, an involvement of radial glial cells was both qualitatively and quantitatively investigated via immunohistochemistry for vimentin, a well‐known marker for reactive gliosis (Figure 6, Figure S3) (Escartin et al., 2021; Zamanian et al., 2012). As described previously (Vanhunsel et al., 2021), Müller glia, the radial glia of the retina, were found to upregulate vimentin expression in both their cell somas and fibers with increasing age. Upon nerve damage, no clear injury‐induced glial response could be observed in young adult or aged killifish (Figure 6a, Figure S3), indicating that Müller glia reactivation likely does not contribute to the regenerative response following ONC in killifish nor to the observed age‐associated regenerative impairment in old fish.

In the optic tectum of uninjured fish, cell bodies of quiescent radial glia are positioned right below the neuronal layer of the PGZ and adjacent to the tectal ventricle. Their individual cytoplasmic processes (fibers) project upwards, protrude in the superficial tectal layers, and terminate as end feet on the pia surface (Lindsey et al., 2019; Than‐Trong & Bally‐Cuif, 2015; Vanhunsel et al., 2021). Similarly, our findings show that in uninjured young female killifish, vimentin staining of diffuse glial end feet was clearly present in the SGC, SFGS, and SO layers of the optic tectum, and to a lesser extent in the radial fibers. Following ONC in 6‐weeks‐old fish, vimentin signal was upregulated in the radial fibers, albeit only at 7 dpi and thus for a short time. Damage to the optic nerve thus seems to elicit a transient and limited response of the radial glia in the young adult killifish tectum. In the older age groups, basal vimentin immunoreactivity was visibly upregulated in the radial fibers, indicative for the occurrence of reactive gliosis in the optic tectum of older fish, as previously observed (Vanhunsel et al., 2021). While the gliotic response of the glial fibers appeared to be transient in 6‐weeks‐old fish, it was prolonged and more pronounced in the older age groups (Figure 6b,c). All in all, these results indicate that radial glia within the tectum of aged female killifish respond differently to ONC, which might contribute to the observed defect in restoration of a functional circuit.

**FIGURE 7:**
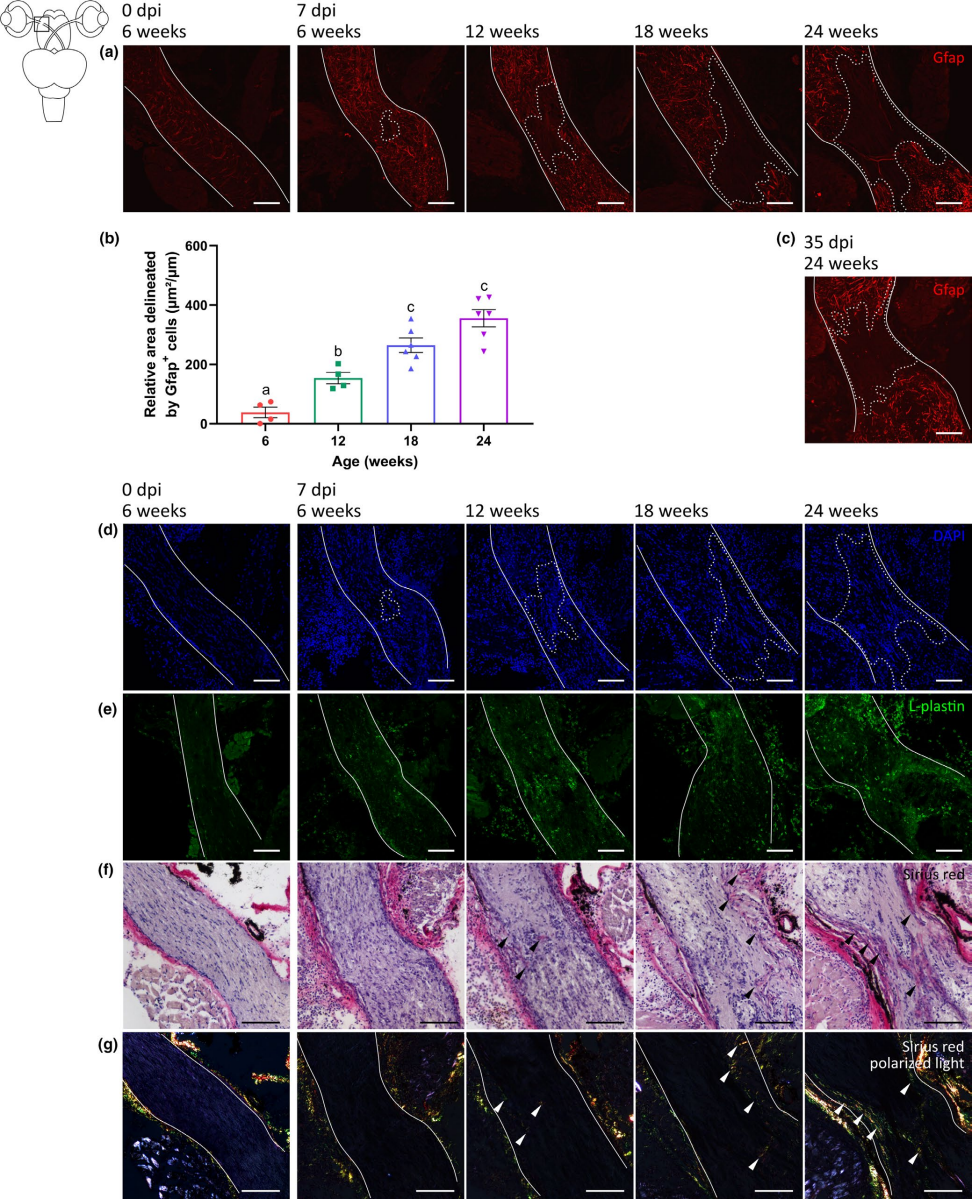
Older killifish form a glial scar following optic nerve crush. (a) Representative microscopic images of Gfap‐stained longitudinal sections of the optic nerve (delineated by white line) of 6‐weeks‐, 12‐weeks‐, 18‐weeks‐, and 24‐weeks‐old killifish subjected to ONC disclose that the Gfap signal surrounding the lesion area intensifies upon ONC as compared to 6‐weeks‐old uninjured fish. Within the lesion area itself, Gfap labeling reveals a loss of astroglial processes in all age groups compared to uninjured 6‐weeks‐old control (dashed line). (b) Compared to 6‐weeks‐old crushed fish, the lesion area, marked by reduced Gfap staining and quantified as the area demarcated by Gfap^+^ labeling, is larger in 12‐weeks‐, 18‐weeks‐, and 24‐weeks‐old fish. *n* = 4–6, data are represented as mean ± SEM, means with a different letter are significantly different (One‐Way ANOVA), see Table S1 for exact *p*‐values. (c) At 35 dpi, this Gfap‐free area is still present in 24‐weeks‐old fish. (d) DAPI staining confirms the presence of cells within the Gfap‐negative area. (e) Immunolabeling for L‐plastin demonstrates an increased number of microglia/leukocytes at the crush site in the optic nerve of all age groups when compared to uncrushed young adult killifish. The immune cell number in the optic nerve of 12‐weeks‐, 18‐weeks‐, and 24‐weeks‐old fish, however, is clearly higher. (f) Sirius red staining of crushed nerves under bright‐field microscopy shows first indications for the presence of a collagen‐rich scar (black arrowheads) in older killifish, which is most pronounced in 18‐weeks‐ and 24‐weeks‐old fish. (g) Sirius red‐stained sections under polarized light disclose the presence of few immature (green) collagen fibers in 12‐weeks‐old fish, and a clear deposition of both immature (green) and mature (red) fibers in 18‐weeks‐ and 24‐weeks‐old killifish (white arrowheads). Scale bar = 100 µm. DAPI, 4′,6‐diamidino‐2‐phenylindole; dpi, days *post*injury; Gfap, glial fibrillary acidic protein; ONC, optic nerve crush

Strikingly, also at the crush site in the optic nerve, we observed signs of gliotic and inflammatory responses in older fish. Following ONC, glial fibrillary acidic protein (Gfap) expression by astrocyte‐like cells surrounding the scar seemed upregulated in all age groups, indicative for reactive gliosis (Escartin et al., 2021; Zamanian et al., 2012). Additionally, at 7 dpi, a Gfap^−^ area could be observed at the ONC injury site in all age groups, reflecting the presence of a glial scar (Figure 7a). Notably, in 12‐weeks‐, 18‐weeks‐, and 24‐weeks‐old fish, this Gfap‐negative area was enlarged as compared to 6‐weeks‐old fish at 7 dpi (Figure 7b) and still present in 24‐weeks‐old fish at 35 dpi (Figure 7c), suggesting more severe damage of the nerve, increased scarring, and/or impaired repopulation by astroglia in older killifish during the repair process (Hilla et al., 2017; Liu et al., 2021; Qu & Jakobs, 2013). Despite reduced Gfap staining within the scar area, this region was not cell‐free (Figure 7d). Rather, in line with findings in rodents (Liu et al., 2021; Qu & Jakobs, 2013), L‐plastin^+^ cells, representing microglia and leukocytes, were observed at the crush site of 6‐weeks‐, 12‐weeks‐, 18‐weeks‐, and 24‐weeks‐old fish as compared to uninjured young adults. In comparison with 6‐weeks‐old injured fish, the number of these immune cells at the lesion site was clearly augmented in the older age groups (Figure 7e), suggestive for a stronger inflammatory response. Interestingly, Sirius Red staining revealed collagen deposition in the glial scar of 12‐weeks‐old fish at 7 dpi, which became more prominent at older age, with a clear presence of both immature (green) and mature (red) collagen fibers in the nerves of 18‐weeks‐ and 24‐weeks‐old fish (Figure 7f,g). Altogether, these data point toward the development of a collagen‐rich glial scar in aged females, which might physically hinder RGC axon regrowth, and thus tectal reinnervation and functional recovery.

## 3 DISCUSSION

Over the last decades, fish models have gained popularity for biological and medical research, also in regenerative studies of the visual system (Bollaerts et al., 2018). While the repair process of optic nerve regeneration has been mapped in detail in zebrafish (Becker & Becker, 2014; Beckers et al., 2019; Bhumika et al., 2015; Diekmann et al., 2015; Kato et al., 2013; Lemmens et al., 2016; McCurley & Callard, 2010; Van houcke et al., 2017; Zou et al., 2013) and to a lesser extent in goldfish (Kato et al., 1999, 2013), this is not the case in the fast‐aging killifish, a more recent teleost model. Using an ONC injury paradigm in female killifish, we show that the time frame of axonal regeneration in young adult killifish (6‐weeks‐old) is very similar to that of zebrafish (Table 1). Upon aging, however, different cellular processes underlying or contributing to the repair process in the visual projection are affected. Remarkably, killifish at old age seem to resemble (young) mammals, with insufficient upregulation of growth‐associated genes, an excessive inflammatory response, glial reactivity, and scarring lying at the basis of the observed regenerative impairment. Note that all data have been acquired using female fish. As sex has been reported to affect the regenerative potential in both rodents and fish, and repair in female rodents/fish is often faster (Blankenhorn et al., 2003; Nachtrab et al., 2011; Xu et al., 2020), it is important to highlight that our results could have differed if male fish had been included in this work.

Strikingly, not all RGCs of young adult killifish regenerate following ONC. Indeed, our data reveal that only 50% of the RGCs initiate regrowth and do so very early after injury. A significant portion (40% of the RGCs) seems to die during the following days/weeks *post*crush, leading to a reduced number of RGCs at 3 weeks *post*injury. These findings highly contrast observations regarding RGC death following ONC in zebrafish, where 99.5% of the RGCs survive (Lemmens et al., 2016; Van houcke et al., 2017; Zou et al., 2013). In line with findings of Wang and colleagues, revealing differences in the genomic responses to fin amputation in killifish and zebrafish (Wang et al., 2020), a distinct RGC intrinsic response to ONC might contribute to this. Indeed, as killifish RGCs only very transiently upregulate growth‐associated genes after ONC compared to zebrafish (Kato et al., 2013; McCurley & Callard, 2010; Van houcke et al., 2017), it seems plausible that RGCs that do not immediately start expressing these genes might not survive. Notably, in addition to RGCs dying, not all RGCs regrow an axon, and a small number (about 10% of the RGCs according to our data) seems to stay alive without reconnecting to their target cells in the optic tectum. Although the morphological and functional heterogeneity of killifish RGCs has not been elucidated, it can be assumed that various subtypes show a different resilience to optic nerve injury, as has been previously shown in rodents (Daniel et al., 2018; De Sevilla Müller et al., 2014; Sánchez‐Migallón et al., 2018; Tran et al., 2019; VanderWall et al., 2020) and recently also suggested for RGCs of zebrafish subjected to optic nerve transection (Chen et al., 2021). Of note, using single‐cell transcriptomics, Kölsch and colleagues identified more than 30 RGC subtypes in the adult zebrafish retina and also revealed to which tectal and extratectal layers these cell types project (Kölsch et al., 2021). A similar study in killifish, whether or not subjected to ONC, would greatly contribute to determining which RGC subtypes survive axonal injury, which ones regenerate or rather remain in the retina without regenerating, and also which visual reflex or behavior the various RGC types subserve.

Another feature that is preserved among species, from teleosts to mammals, is the remarkable plasticity of the visual system. Indeed, with only ~50% of the RGCs regrowing an axon, young adult females are still able to completely reinnervate their optic tectum and restore their optokinetic response. This finding suggests tectal plasticity and thus that, besides RGC axon outgrowth, also axonal branching contributes to functional recovery after optic nerve injury. Note that while our results reveal complete reinnervation of the optic tectum upon ONC in 6‐weeks‐old fish, we cannot exclude inadequate innervation of other arborization fields and thus incomplete restoration of the full visual circuit. As the swimming angle of some young adult fish remained different from baseline values, this might be the case for the suprachiasmatic nucleus, which is suggested to be involved in the dorsal light reflex (Nevin et al., 2010). It is, however, important to know that, at least in zebrafish, the axons that reach the other arborization fields are almost all collaterals from axons that run toward the optic tectum (97% of the axons project to the optic tectum and half of them form collaterals; 3% extend to the pretectum and do not reach the optic tectum) (Kölsch et al., 2021; Nevin et al., 2010; Robles et al., 2014), implying that reinnervation of the tectum is a good readout for arborization of the other areas. Also, within this study, we determined synaptic repair and restoration of primary vision after optic nerve damage, but did not study the complete retinotopic reestablishment and synaptic refinement. While reflexes such as the optokinetic response and dorsal light reflex are considered good readouts for functional recovery from ONC (Neuhauss, 2010; Nevin et al., 2010), electrophysiology and more complex visual behavior tests (*e*.*g*., optomotor response and chasing behavior) are required to fully explore this (Becker & Becker, 2014; Bollaerts et al., 2018; McGinn et al., 2018).

**FIGURE 8:**
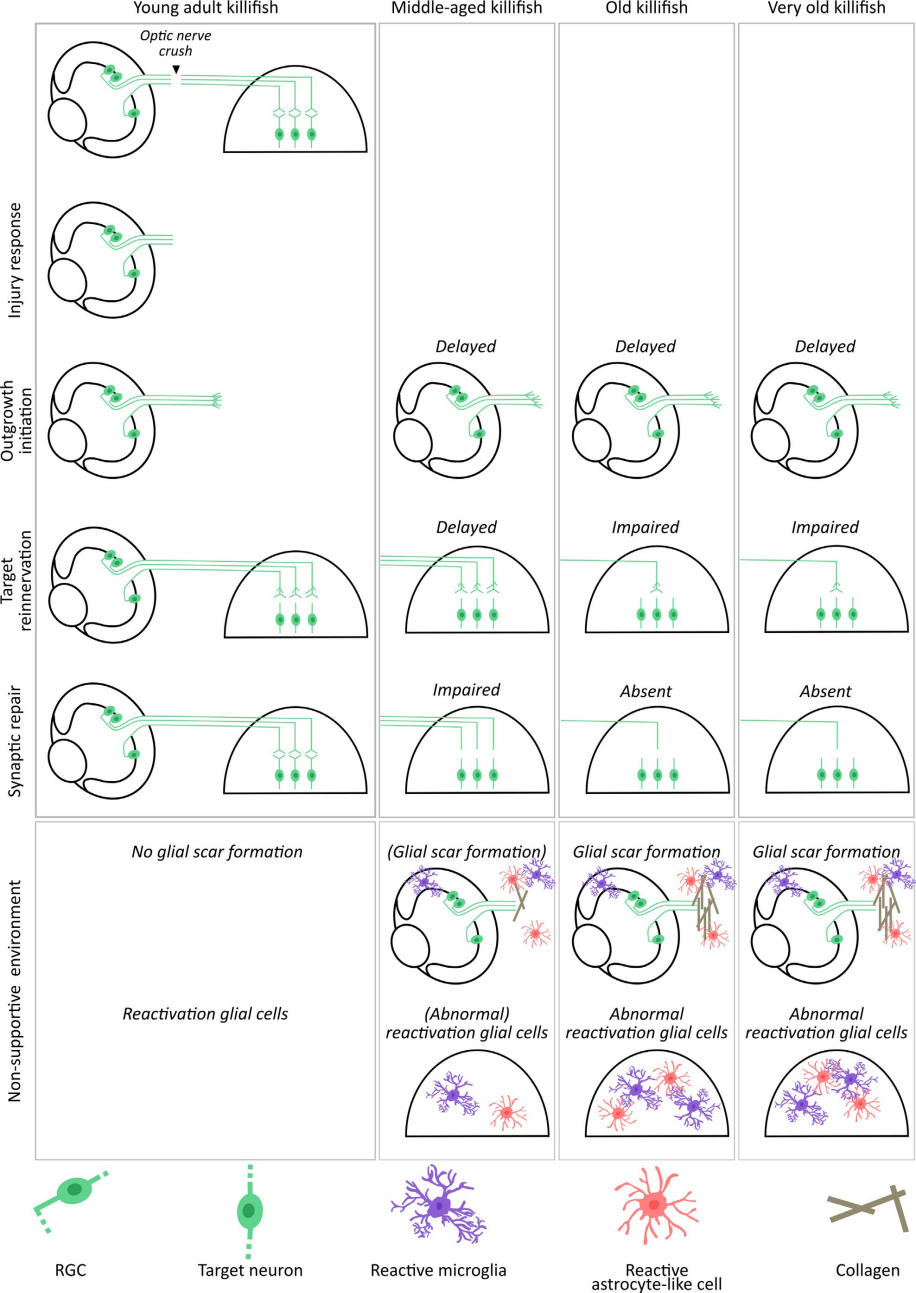
Summary scheme: Aging affects different phases of the regenerative process in different killifish age groups. Schematic representation of the age‐dependent decline in regenerative ability of 6‐weeks‐, 12‐weeks‐, 18‐weeks‐, and 24‐weeks‐old killifish. While young adult killifish are capable of functionally recovering from ONC, regeneration fails at different phases of the repair process in older fish. All older killifish suffer from a decreased intrinsic growth capacity of RGCs (green), resulting in a delay of axon outgrowth initiation. Despite a retarded but complete tectal reinnervation, 12‐weeks‐old fish do not fully recover from ONC, suggesting a defect in synaptic repair. RGCs of 18‐weeks‐ and 24‐weeks‐old fish fail to completely reinnervate the optic tectum. Also synaptic repair is absent, resulting in old and very old fish not recovering their sight. Additionally, also age‐associated alterations in the cellular environment seem to contribute to impaired axonal regeneration in middle‐aged, old, and very old fish. Abnormal reactivation of glial cells in the retina, tectum, and nerve, including microglia (purple) and/or astrocyte‐like cells (red), seems to result in a more chronic inflammatory response, defects in synaptic repair and the formation of a collagen‐rich (brown) glial scar, thereby contributing to the impaired axonal regeneration in the aged killifish retinotectal system. ONC, optic nerve crush; RGC, retinal ganglion cell

Despite the universal acceptance that RGCs of teleost fish have an impressive intrinsic regenerative ability and do not suffer from an overall inhibitory environment (Becker & Becker, 2007, 2014), this might not hold true for aged fish species. In fact, our data indicate that old killifish resemble (young) mammals, in which both intrinsic and extrinsic factors have been shown to inhibit long‐range regrowth of RGC axons in the optic nerve (Figure 8) (Bollaerts et al., 2018; Curcio & Bradke, 2018; Uyeda & Muramatsu, 2020; Yin et al., 2019). In middle‐aged fish (12 weeks), the intrinsic growth capacity of RGCs is decreased, leading to a delayed yet eventually full axonal regeneration, comparable to what has been observed in aged zebrafish (Van houcke et al., 2017). Given that, just like in young adult fish, only ~50% of the RGCs regenerated an axon, this shows that also a certain level of plasticity still exists in these middle‐aged fish. Of note, as killifish body length increases with about 32% between 6 weeks and 12 weeks of age (Vanhunsel et al., 2021), one could assume that the delay in tectal reinnervation might (in part) be attributable to axons having to overcome longer distances. However, also when studying axonal outgrowth initiation, during which the retrograde biocytin tracer was always placed at the same distance from the ONC site, a retardation was observed, which somewhat counters this alternative explanation. Interestingly, and in contrast to older zebrafish (Van houcke et al., 2017), 12‐weeks‐old killifish do not completely reconstitute their sight. While partially regaining visual acuity, they do not recover the dorsal light reflex at all. Likely, in addition to the perceived decrease in intrinsic growth ability of RGC axons, also synaptic repair and refinement, and/or reinnervation of additional target areas in the brain that are also involved in this reflexive behavior, might be impaired in middle‐aged fish, altogether leading to the observed incomplete recovery of visual function (Figure 8).

In contrast to middle‐aged fish, 18‐weeks‐ and 24‐weeks‐old fish do not completely reinnervate their optic tectum. Differential expression levels of *gap43* and *tuba1a* in 18‐weeks‐ and 24‐weeks‐old fish *versus* 12‐weeks‐old fish suggest that these older fish might not express sufficient growth‐associated genes, leading to impaired axonal outgrowth initiation. As the number of regrowing RGCs in 18‐weeks‐old fish is comparable to that of 12‐weeks‐old fish at 21 dpi, some axons might still pass the site of biocytin tracer placement in these old fish, yet next stop elongating because of scarce expression of growth‐associated genes, resulting in axons barely or never reaching the optic tectum and thus the absence of synapse reformation. For 24‐weeks‐old fish, an even more reduced RGC intrinsic outgrowth ability seems to result in only ~30% of the RGCs elongating an axon at 21 dpi, causing even less RGC axons to arrive in the optic tectum and failure to form synapses. Altogether, flawed axonal outgrowth initiation and elongation can be assumed to underlie the incomplete tectal reinnervation, the absence of synaptic repair, and ultimately the lack of sight recovery in old and very old fish (Figure 8). These results are in line with data reporting on fin regeneration in killifish, in which a delayed initiation of regenerative processes is suggested to underlie the finding that old fish never reach their original fin size following amputation (Wendler et al., 2015). RGC intrinsic pathways known to control RGC growth are the Jak/Stat, MAPK/ERK, and PI3K/Akt pathways. Targeting these pathways—*for example*, using CRISPR/Cas9 technology—via, among others, activation of the mechanistic target of rapamycin (mTOR) pathway and deletion of Krüppel‐like family of transcription (KLF), phosphatase and tensin homolog (PTEN), and/or suppressor of cytokine signaling 3  (SOCS3) (Andries et al., 2020; Benowitz et al., 2017; Chung et al., 2020; Elsaeidi et al., 2014; Fischer & Leibinger, 2012; Yin et al., 2019) would provide novel mechanistic insights on how to stimulate RGC axonal regrowth in old killifish.

In addition to a declined neuron‐intrinsic growth potential, also a non‐supportive extracellular milieu seems to contribute to the observed delay and/or impairment of optic nerve regeneration in older female killifish. We show that aging affects the contribution of different glial cell types at different locations in the visual projection following ONC: in the retina, optic nerve, and optic tectum. First of all, in the retina, immune cells respond differently to ONC in aged killifish when compared to young adult killifish. Indeed, the chronic low‐grade inflammatory status that we previously noticed in the uninjured killifish visual system (Vanhunsel et al., 2021) seems to result in a dysregulated inflammatory response in the killifish retina following ONC, similar to what has been observed in aged mammals (Cantuti‐Castelvetri et al., 2018; Damani et al., 2011; Wasserman et al., 2008) and in aged zebrafish (Münzel et al., 2014; Van houcke et al., 2017). In the young adult killifish retina, only a transient immune response is observed following ONC, with a clear increase in microglia/leukocyte cell number at 2 dpi that returned to baseline around 14 dpi. This transient acute reaction is also described in young adult zebrafish, where it is suggested to support optic nerve regeneration (Bollaerts et al., 2017, 2019; Chen et al., 2021; Van houcke et al., 2019; Zou et al., 2013). In mammals, inducing a controlled immune response in the retina also improves the regenerative outcome following ONC (Bollaerts et al., 2017; Yin et al., 2019). Chronic inflammation, however, has been demonstrated to coincide with RGC death, both in fish and in mammals (Bollaerts et al., 2017; Zou et al., 2013). Moreover, blocking neuroinflammation or depleting immune cells in zebrafish subjected to optic nerve transection was shown to rescue RGC survival (Chen et al., 2021). As such, the observed extensive and/or prolonged immune response following ONC in the aged killifish retina might change the immune environment toward one that resembles chronic inflammation, with altered inflammatory cell numbers and phenotypic states, and contribute to the observed regenerative decline after nerve injury in older fish. Also abnormal activation or functioning of astrocyte‐like cells in the retina (*i*.*e*., Müller glia) might affect the recovering ability of aged killifish from ONC. Upon stress, injury (including ONC), or disease in the mammalian retina, Müller glia typically undergo reactive gliosis to prevent further damage, promote repair, and limit tissue remodeling (Bringmann & Wiedemann, 2011; Sun et al., 2017). In zebrafish, only extensive retinal damage, such as injury induced by exposure to neurotoxins, by mechanical damage, or by intense light, but not ONC, results in this response (Lahne et al., 2020; Thomas et al., 2016). Interestingly, despite the observed cell death following ONC in the killifish, and thus the possible presence of damage signals, no gliotic response was found at any age. While a more detailed characterization is still needed, this suggests that even in old killifish the extent of RGC loss upon ONC does not elicit reactive gliosis of Müller glia.

In contrast, an augmented reactivation of astrocyte‐like cells (radial glia) was observed in the optic tectum of aged females. Since astrocyte‐like cells are known to play a role in synapse formation, maturation, elimination, and synaptic signal transmission, an abnormal response following ONC might affect synapse reformation and eventual circuit repair (Burda et al., 2016; Chung et al., 2015; Escartin et al., 2019). Indeed, following injury, astrocyte reactivation has been observed to be more pronounced and prolonged in aged animals (Clarke et al., 2018; Geoffroy et al., 2016; Heimann & Sirko, 2019; Kyrkanides et al., 2001; Lane et al., 2007; Sandhir & Berman, 2010), which corroborates our findings and supports the hypothesis that enhanced glial reactivity prevents correct formation of functional synapses in the aged killifish. Besides actively participating in immune responses, also microglia promote the formation of synapses and circuit refinement by phagocytosing excessive and immature synapses (Bar & Barak, 2019; Jurgens & Johnson, 2012; Marinelli et al., 2019; Ramirez et al., 2017). Just like in the retina, the presence of immune cells is prolonged in the tectum following ONC. This extended inflammatory response in the tectum might therefore result in an increased engulfment of (newly formed) synapses in old killifish and in that way prevent synapses from being properly reformed in aged fish (Lui et al., 2016).

In addition to altered glial responses in the retina and/or tectum of old killifish subjected to ONC, our data also revealed an excessive presence and/or reactivity of microglia/infiltrating leukocytes and astrocyte‐like cells at the injury site in older killifish when compared to young adults. These cells constitute two major components of the glial scar that is formed in rodents following nerve and spinal cord injury (Bellver‐Landete et al., 2019; Bradbury & Burnside, 2019; Qu & Jakobs, 2013; Yang et al., 2020). Moreover, although no major scarring was observed in young adult killifish, similar to what is reported in young adult zebrafish subjected to ONC (Becker & Becker, 2007), we observed a large Gfap‐negative scar area and collagen deposition in 12‐weeks‐, 18‐weeks‐, and 24‐weeks‐old fish, with the most prominent scarring observable at the optic nerve lesion site in old and very old (the oldest) fish. These results are in agreement with findings of Van houcke and coworkers, who reported signs of glial scarring upon stab‐wound lesion in the aged killifish telencephalon (Van houcke et al., 2021). Altogether, abnormal activation and/or presence of glial cells might thus trigger the formation of a collagen‐rich glial scar at the site of nerve crush, which has been shown to both physically and chemically prevent outgrowth of axons in mammals (Bradbury & Burnside, 2019; Kawano et al., 2012; Yang et al., 2020) and thus also likely inhibits nerve regeneration in older killifish.

In conclusion, in female killifish, the regenerative process following ONC seems to resemble that of zebrafish at young age, but switches to a more mammalian‐like phenotype at old age. Depending on the age, regeneration fails at different stages of the regenerative process. Our results show that aged females are suffering from a declined intrinsic growth capacity of RGCs, contributing to a delay/absence in tectal reinnervation and synaptogenesis, and the subsequent lack of functional recovery. In addition to this diminished intrinsic growth ability, our data suggest that also alterations in the extrinsic RGC environment likely hold back axonal outgrowth initiation and elongation, target reinnervation, synaptic reformation, and finally reconstitution of vision. Indeed, abnormal reactivation of glial cells, *that is*, microglia and astrocyte‐like cells, seems to result in the formation of a glial scar and/or failure to properly reform synapses, which might underlie the defective optic nerve regeneration in older fish. These findings highlight the importance of the neuron‐glia crosstalk for CNS repair, and how age‐related changes in their communication might affect the regenerative ability. Additional research, however, is needed to further disentangle the exact contribution of the different cellular players and the underlying molecular mechanisms that prevent successful regeneration in an aged environment. Preventing an excessive immune response—by for example administering anti‐inflammatory drugs, inhibiting cytokine signaling or blocking the NF‐kB pathway (Andries et al., 2020; Cellerino & Ori, 2017; Chen et al., 2021; Keatinge et al., 2021; Luo et al., 2007; Wei et al., 2020)—, the abnormal reactivity of astrocyte‐like cells—by for example impeding the upregulation of Gfap or vimentin (Hippert et al., 2021)—, or the formation of a glial scar—by administering chrondroitinases (Bradbury et al., 2002; Kim et al., 2006)—would provide important information on how to modulate scar formation and/or synaptogenesis to finally promote axonal regeneration and functional recovery in old killifish. Additionally, figuring out which exact molecules and signaling pathways are differentially expressed/regulated in young and old killifish, but also in (aged) killifish, zebrafish, and mammals would be of great value for the identification of regenerative molecules or even rejuvenating drugs. All in all, we position the killifish as a unique model to study the underlying mechanisms of injury‐induced axonal regeneration and synaptic repair in an aged setting that can help to shed light on regenerating mammalian visual systems and possibly other tissues resistant to regeneration.

## 4 EXPERIMENTAL PROCEDURES

### 4.1 Fish and husbandry

For all experiments, adult fish of the *Nothobranchius furzeri* GRZ‐AD inbred strain were used. Fish were fed twice a day with frozen red bloodworms (*Chironomidae)* and maintained under standardized conditions at a temperature of 28.3°C, conductivity of 600 µS, and pH 7, on a 12‐h light/12‐h dark cycle. Fish were kept in 3,5 L ZebTec tanks (Tecniplast), each housing one male and three females. Considering the size difference of male and female fish, only female fish were used for experiments. The age groups chosen to study optic nerve regeneration represent young adult, middle‐aged, old, and very old fish, with an age of 6 weeks, 12 weeks, 18 weeks, and 24 weeks, respectively, and each contain equally sized fish. All animal experiments were approved by the KU Leuven Animal Ethics Committee and carried out in strict accordance with the European Communities Council Directive of 20 October 2010 (2010/63/EU).

### 4.2 Optic nerve crush

An ONC was performed as described for zebrafish (Beckers et al., 2019; Bollaerts et al., 2019; Van houcke et al., 2017). Briefly, killifish were anesthetized by submersion in 0.03% Tris‐buffered tricaine (MS‐222, Sigma‐Aldrich) and put under a dissection microscope with their left side facing upwards. Next, the connective tissue surrounding the left eye was cut to be able to lift the eyeball out of its orbit, thereby exposing the optic nerve and ophthalmic artery. After carefully placing sterile forceps (Dumont No. 5, FST) around the left optic nerve, a crush of 10 s at 0.5 mm distance from the optic nerve head was performed. Note that the duration of the crush and distance from the optic nerve head were kept fixed for all age groups and fish sizes. The ONC was considered successful when a clear gap inside the translucent nerve sheath appeared and damage to the ophthalmic artery was avoided. Fish were returned to system water to recover. For evaluation of the optokinetic response, a bilateral ONC was performed to prevent an effect from binocular vision (Van houcke et al., 2017).

### 4.3 RNA isolation and real‐time quantitative polymerase chain reaction (RT‐qPCR)

To measure the expression levels of *gap43* and *tuba1a* in the retina of young adult, middle‐aged, old, and very old fish at 0, 3, 7, and 14 dpi, and of pro‐inflammatory cytokines *il*‐*1β*, *tnf*, *il*‐*6*, and *il*‐*8* in the retina and tectum of young adult and very old fish at 0, 2, 7, and 21 dpi, RT‐qPCR was performed. Whole retinas and/or tecti were quickly dissected on ice and then pooled per two. Next, the pooled tissues were homogenized in Tri reagent (Sigma‐Aldrich), and total RNA was extracted by NucleoSpin RNA isolation kit (Machery‐Nagel). Using oligo dT primers and Superscript III reverse transcriptase (Invitrogen), RNA was reverse‐transcribed to cDNA. The RT‐qPCR reactions were done using SYBR Green master mix (BioRad) and a CFX96 Touch Real‐Time detection system (BioRad). All samples were run in duplicate, at least three independent samples per experimental condition were analyzed, and an annealing temperature of 60°C was used. Using qBase software (Biogazelle), reference genes were selected and gene expression values analyzed. All used primer sequences are catalogued in Table S2. Finally, values are put relative to those of uninjured control fish and normalized against the geometric mean of our reference genes.

### 4.4 Retrograde tracing and quantification of the number of regenerating RGCs

The number of regenerating RGCs was evaluated using retrograde biocytin tracing toward the retina. The tracing was performed as described previously (Van houcke et al., 2017). Briefly, fish were anesthetized in 0.03% Tris‐buffered tricaine and positioned under a dissection microscope, with their left side facing upwards. Next, the connective tissue surrounding the eyeball was cut. After lifting the eye out of its socket, the left optic nerve was cut between the crush site and the optic chiasm, and the biocytin tracer was placed on the proximal nerve end. Subsequently, the fish were revived for 3 h, allowing the biocytin to be taken up by the regrowing axons and retrogradely transported toward the RGC cell bodies in the retina. Following tracing, the fish were euthanized and transcardially perfused with phosphate‐buffered saline (PBS; 0.01 M, pH 7.4), followed by 4% paraformaldehyde (PFA) in PBS. Thereafter, the left eye was dissected and *post*‐fixed overnight in 4% PFA in PBS. After processing for cryosectioning, 10‐µm thick serial sagittal sections of the eye were made. Biocytin in the RGC soma was visualized using Alexa‐coupled streptavidin (1:200; Invitrogen). Images were acquired using an Olympus FV1000 confocal microscope at 20x magnification. Biocytin^−^/DAPI^+^ and biocytin^+^/DAPI^+^ cells were counted on at least three central retinal sections and averaged per fish. All experimental groups included a minimum of six fish. The obtained values for injured fish were plotted as absolute numbers or as a percentage of biocytin^+^/DAPI^+^ cells relative to the total number of biocytin^+^/DAPI^+^ cells in the GCL of uninjured control fish of the same age group, which was set as a 100% reference value.

### 4.5 Anterograde tracing and quantification of tectal (re)innervation

To investigate tectal (re)innervation in both uninjured fish and fish subjected to ONC, anterograde tracing was performed using biocytin tracer, all as previously described (Beckers et al., 2019; Bhumika et al., 2015; Bollaerts et al., 2019; Van houcke et al., 2017). Briefly, fish were anesthetized in 0.03% Tris‐buffered tricaine and positioned under a dissection microscope, with their left side facing upwards. After cutting the connective tissue surrounding the eyeball and lifting the eye out of its socket, the left optic nerve was cut between the optic nerve head and the crush site. Subsequent to applying the tracer on the distal optic nerve end, the fish were revived for 3 h, allowing the tracer to be taken up and anterogradely transported along the RGC axons that are regrowing toward the optic tectum. Ultimately, the fish were euthanized in 0.1% Tris‐buffered tricaine and transcardially perfused as described above. The brains were dissected, fixed overnight in 4% PFA, and processed for serial coronal vibratome (sections of 50 µm) and cryostat sectioning (sections of 10 µm). On vibratome sections, biocytin was visualized by means of a Vectastain ABC kit (Vector laboratories) and diaminobenzidine (DAB) as a chromogen. Next, brain sections were mounted on gelatin‐coated glass slides and counterstained with neutral red solution (Sigma‐Aldrich) to allow for the identification of specific brain nuclei. The (regenerated) axons were also visualized on cryosections using an Alexa‐coupled streptavidin (1:200; Invitrogen), followed by a nuclear stain using DAPI (1:1000; Sigma‐Aldrich) on cryosections. Sections containing the central optic tectum were identified based on the presence of certain brain nuclei using the killifish brain atlas (D’Angelo, 2013). Next, bright‐field images of the midcoronal tectum at 10× magnification were obtained using a Zeiss Imager Z1 microscope and fluorescent images were acquired using an FV1000 confocal microscope (Olympus) at 20×. (Re)innervation of the optic tectum was quantified via an in‐house‐developed Fiji script, measuring both the biocytin^+^ area via a preset threshold and the total tectal RGC innervation area, comprising the SFGS and SO tectal layers. Axonal density levels, being the ratio of the biocytin^+^ area to the total RGC innervation area, were determined in at minimum four fish per experimental group on at least three sections that contained the central optic tectum. For every age group, axonal density levels of uninjured fish were set as 100%. Reinnervation values of injured fish were then expressed as a percentage relative to uninjured control fish of the same age group.

### 4.6 (Immuno)histochemistry

For immunohistochemistry, fish were euthanized in 0.1% Tris‐buffered tricaine and perfused with PBS, followed by 4% PFA in PBS. Eyes, brains, and entire visual systems of fish of various ages and at different days *post*‐ONC were dissected. Next, the tissues were *post*‐fixed for 1 h (eyes and brains) or 6 h (visual systems) at room temperature, or overnight at 4°C (eyes for L‐plastin staining) in 4% PFA and processed for cryostat sectioning of 10‐µm sections (sagittal retinal sections, coronal brain sections, and frontal visual system sections, which result in longitudinal sections of the optic nerve). The following primary antibodies were used: rabbit anti‐activated‐caspase‐3 (1:70; BioVision), rabbit anti‐Gfap (1:200; DAKO), rabbit anti‐L‐plastin (1:500; Genetex), mouse anti‐vimentin (1:400, Sigma‐Aldrich), and mouse Znp‐1 (1:500; DSHB). More detailed antibody information is summarized in Table S3. Primary antibodies were detected using secondary antibodies conjugated with Alexa or with horseradish peroxidase (Dako) combined with the TSA^TM^ FT system (PerkinElmer). Visualization was performed using an Olympus FV1000 confocal microscope at 20× magnification.

Activated‐caspase‐3^+^ cells in the GCL were counted over the entire length of a retinal section on at least three central retinal sections per fish (max 240 µm from the section containing the optic nerve head) and then averaged per fish. All experimental groups encompassed a minimum of three fish. The Znp‐1, L‐plastin, and vimentin stainings were quantified by defining the immunopositive area within a fixed region of the central retina (~0.04 mm²) and/or tectum (~0.12 mm² for the L‐plastin staining, covering all layers in the optic tectum; 0.03 mm² for the Znp‐1 and vimentin stainings, covering the SFGS layer or the SGC and S/S layers of the optic tectum, respectively). The area occupied by the immunopositive signal was determined via automated thresholding in Fiji (Triangle), which was then divided by the predefined area. Quantification was performed on at least two midsagittal or midcoronal sections per fish and then averaged. At least three fish were evaluated per condition. The size of the glial scar was evaluated as the region delineated by Gfap^+^ cells, followed by normalization to the width of the nerve at the site of injury. This scar area was quantified on at least two nerve sections and then averaged per fish. Per condition, at minimum four fish were analyzed.

For Sirius Red staining of the visual systems, sections were incubated with Picric acid (Sigma) with direct red (3 mg/300 ml) for 1 h and subsequently in HCl (0.01N) for 2 min. Next, the sections were stained for 3 min with hematoxylin according to Mayer, followed by a dehydration series and mounting with Neomount. Pictures of the optic nerve were taken under bright‐field and polarized light using a Leica imager at a 20x magnification.

### 4.7 Optokinetic response test

To assess recovery of primary vision, adult killifish of 6‐weeks‐, 12‐weeks‐, 18‐weeks‐, and 24‐weeks‐old were subjected to an optokinetic response test (Van houcke et al., 2017) at 0, 2, 4, 7, 10, 14, 18, 35, 42, 55, and 60 days following bilateral ONC. First, fish were anesthetized briefly in 0.03% Tris‐buffered tricaine. After positioning them in a custom‐made glass chamber, a continuous water flow was provided, allowing the fish to reawaken and breathe while being immobilized. The glass chamber was then placed in an OptoMotry device (Cerebral Mechanics) for assessment of visual acuity. The maximal spatial frequency provoking an optokinetic reflex of each fish was determined, while velocity and contrast were kept fixed at 15 deg/s and 100%, respectively. Each trial was initiated with a spatial frequency of 0.02 (c/d), which increased stepwise following a staircase model. One day on beforehand, fish were allowed for an adaptation period in the system. For every age group, the optokinetic reflex of at least six fish was followed up (with exception of 6‐weeks‐old fish at 10 dpi (*n* = 5) and of 24‐weeks‐old fish at 18 dpi (*n* = 4)).

### 4.8 Dorsal light reflex

Another measure for evaluating a regain of visual function is the dorsal light reflex. Upon unilateral ONC, fish have been shown to swim in a slightly tilted position, which gradually reverses as regeneration progresses (Diekmann et al., 2015; Lindsey & Powers, 2007; Mensinger & Powers, 2007). Therefore, to determine vision recovery, the degree of tilting was determined at 0, 1, 4, 7, 10, 14, 21, 25, 35, 42, 55, and 65 dpi. Briefly, fish were put in a 2.7 × 17.5 cm container containing 400 ml of system water. First, an adaptation period of 5 min was allowed, after which the fish were recorded for about 2 min in order to capture at least three swims in which the fish body was positioned straight toward the camera, with the frontal body axis parallel to the base of the container. In separate frames of these straight swims, the angle between the fish body position (imaginary line between the fish's eyes) and the horizon was determined using Fiji. Per fish and per time point, at least three frames were analyzed to obtain an average swimming angle per fish. The dorsal light reflex of at least five fish per age group was determined (except for 18‐weeks‐old fish at 0 dpi (*n* = 4), and 24‐weeks‐old fish at 55 dpi (*n* = 3) and 65 dpi (*n* = 1)).

### 4.9 Experimental design and statistical analysis

All values are represented as mean ± standard error of mean (SEM), except the RT‐qPCR data in which mean fold change values relative to uninjured values ± SEM are shown. The value of *n* depicts the number of animals used per condition, thus biological replicates, and is indicated in the respective figure caption. Statistical analyses were performed using GraphPad Prism 8 software. All raw data were first tested for parallel equal variance and normal distribution, using the Brown–Forsythe and Kolmogorov–Smirnov test, respectively, and these assumptions were met in all cases. Significance of intergroup differences was evaluated using one‐ or two‐way ANOVA followed by a Tukey *post hoc* test. Statistically significant differences (*p*‐value <0.05) between various experimental groups are indicated using different letters, *that is*, if experimental groups do not have a similar or shared letter, the mean values of these groups were found statistically different from each other.

## CONFLICT OF INTEREST

The authors declare that they have no conflicts of interest.

## AUTHORS CONTRIBUTIONS

SV performed conceptualization and experiments, analyzed data, and drafted the manuscript. SB performed experiments and edited the manuscript. AB and LDG supported experiments and edited the manuscript. IE and TVB supported experiments. LM performed conceptualization, supervised the work, and reviewed the manuscript.

## Supporting information

Fig S1

Fig S2

Fig S3

Table S1‐S3

## Data Availability

The data sets generated and analyzed during the current study are available from the corresponding author upon reasonable request.
