# Supplementary figures and images for: The age factor in optic nerve regeneration: Intrinsic and extrinsic barriers hinder successful recovery in the short‐living killifish
Source: Aging Cell. 2021 Dec 19;21(1):e13537. doi: 10.1111/acel.13537 (PMC8761009; doi:10.1111/acel.13537)

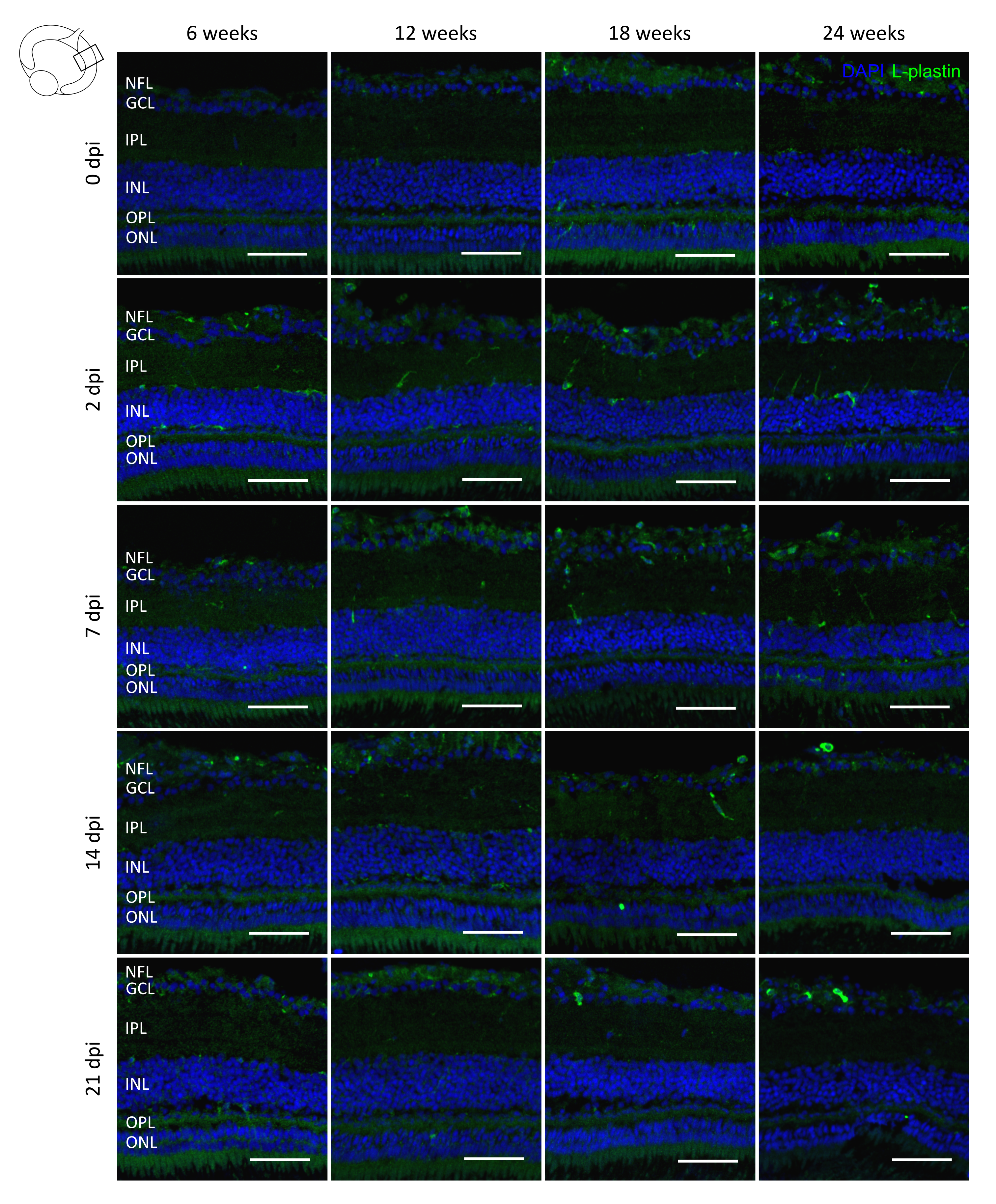

Supplement: Supplementary file 1 — Fig S1 [file ACEL-21-e13537-s004.png]

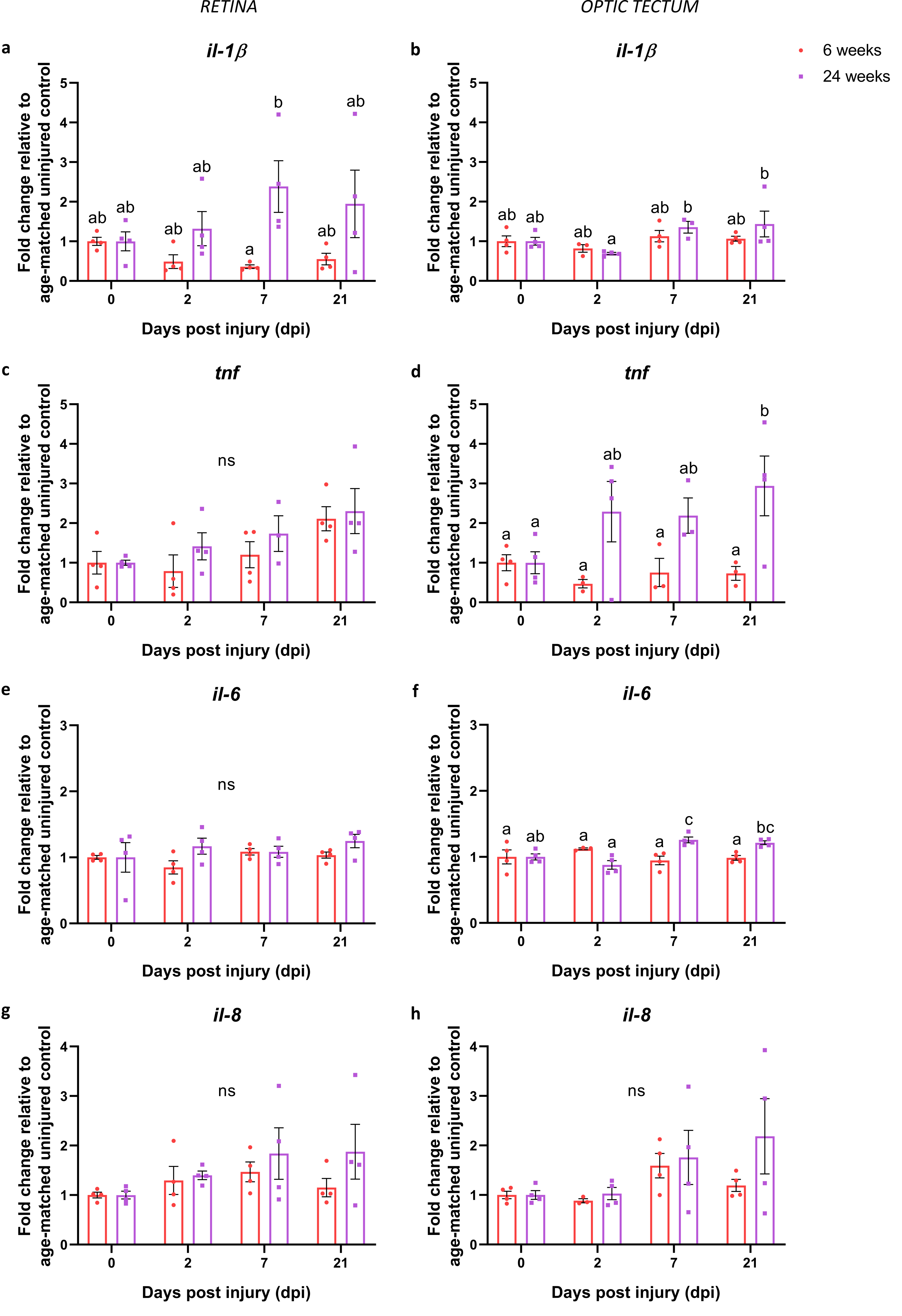

Supplement: Supplementary file 2 — Fig S2 [file ACEL-21-e13537-s002.png]

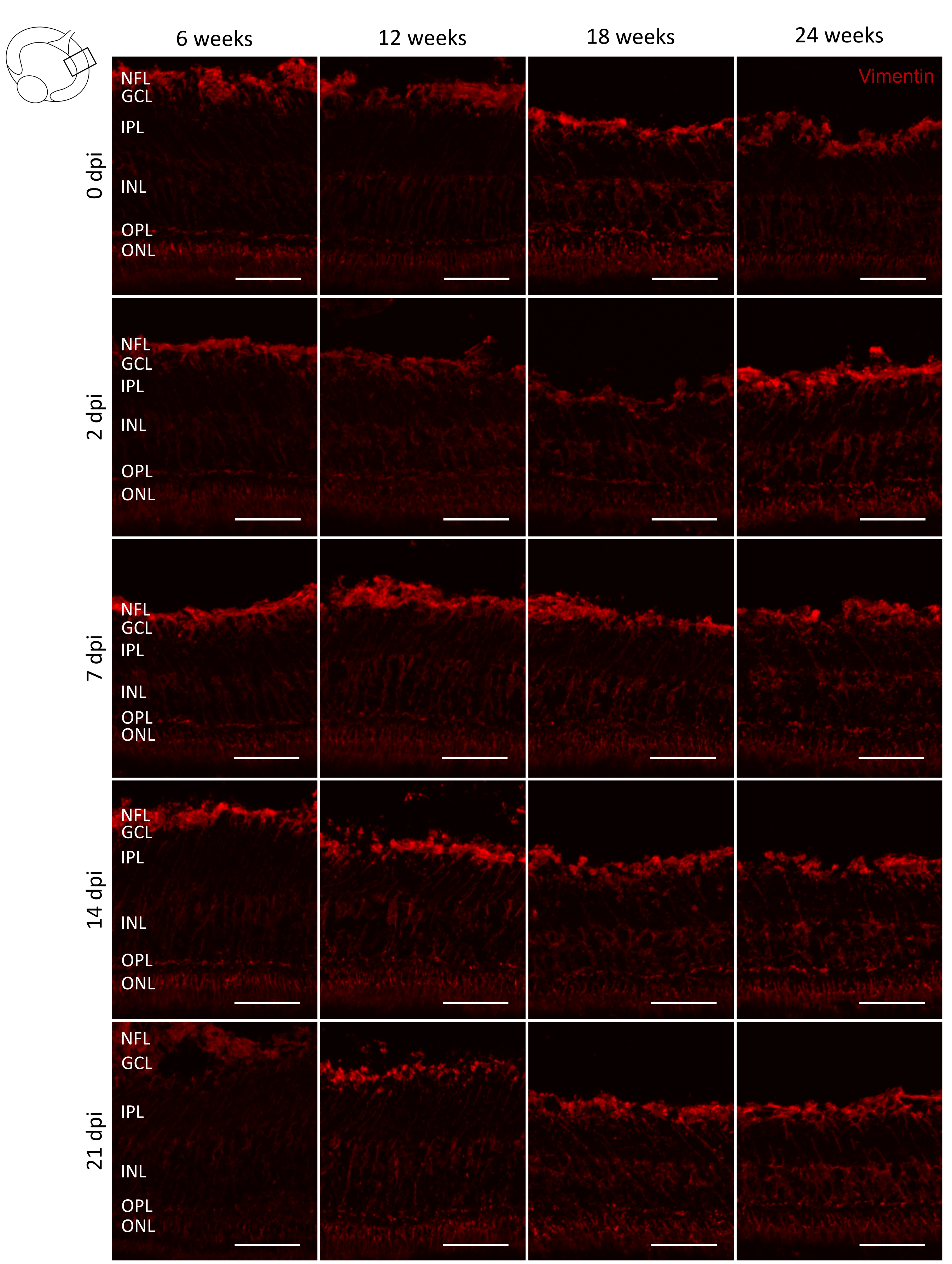

Supplement: Supplementary file 3 — Fig S3 [file ACEL-21-e13537-s003.png]
