# Supplementary material for: The age factor in optic nerve regeneration: Intrinsic and extrinsic barriers hinder successful recovery in the short‐living killifish
Source: Aging Cell. 2021 Dec 19;21(1):e13537. doi: 10.1111/acel.13537 (PMC8761009; doi:10.1111/acel.13537)
Supplement: Supplementary file 4 — Table S1‐S3 [file ACEL-21-e13537-s001.docx]

**Table S1: List of all p-values obtained following statistical testing**

| **Figure 1a** |  | **Figure 1b** |  |  |  |
| --- | --- | --- | --- | --- | --- |
| **One-way ANOVA** |  | **One-way ANOVA** |  |  |  |
| 0 dpi vs. 3 dpi | <0,0001 | 0 dpi vs. 3 dpi | 0,0018 |  |  |
| 0 dpi vs. 7 dpi | 0,5946 | 0 dpi vs. 7 dpi | 0,2888 |  |  |
| 0 dpi vs. 14 dpi | 0,8918 | 0 dpi vs. 14 dpi | 0,7938 |  |  |
| 3 dpi vs. 7 dpi | <0,0001 | 3 dpi vs. 7 dpi | 0,0201 |  |  |
| 3 dpi vs. 14 dpi | <0,0001 | 3 dpi vs. 14 dpi | 0,0054 |  |  |
| 7 dpi vs. 14 dpi | 0,2661 | 7 dpi vs. 14 dpi | 0,7501 |  |  |
| **Figure 1d** |  |  |  |  |  |
| **Two-way ANOVA** | **Total** | **Bioc^-^/DAPI^+^** | **Bioc^+^/DAPI^+^** |  |  |
| 0 dpi vs. 2 dpi | 0,9217 | <0,0001 | <0,0001 |  |  |
| 0 dpi vs. 7 dpi | <0,0001 | 0,9999 | <0,0001 |  |  |
| 0 dpi vs. 14 dpi | <0,0001 | 0,9993 | <0,0001 |  |  |
| 0 dpi vs. 21 dpi | <0,0001 | 0,2082 | <0,0001 |  |  |
| 2 dpi vs. 7 dpi | <0,0001 | <0,0001 | 0,0002 |  |  |
| 2 dpi vs. 14 dpi | 0,0004 | <0,0001 | <0,0001 |  |  |
| 2 dpi vs. 21 dpi | <0,0001 | <0,0001 | 0,012 |  |  |
| 7 dpi vs. 14 dpi | 0,9964 | >0,9999 | 0,9917 |  |  |
| 7 dpi vs. 21 dpi | 0,0387 | 0,3223 | 0,8678 |  |  |
| 14 dpi vs. 21 dpi | 0,0188 | 0,4031 | 0,6475 |  |  |
|  | **0 dpi** | **2 dpi** | **7 dpi** | **14 dpi** | **21 dpi** |
| Bioc^+^/DAPI^+^ vs. Total | <0,0001 | <0,0001 | <0,0001 | <0,0001 | <0,0001 |
| Bioc^+^/DAPI^+^ vs.  Bioc^-^/DAPI^+^ | 0,8222 | <0,0001 | <0,0001 | 0,0005 | 0,0019 |
| Total vs. Bioc^-^/DAPI^+^ | <0,0001 | 0,3937 | <0,0001 | <0,0001 | <0,0001 |
| **Figure 2a** |  |  |  |  |  |
| **Two-way ANOVA** | **6w-old** | **12w-old** | **18w-old** | **24w-old** |  |
| 0 dpi vs. 2 dpi | <0,0001 | <0,0001 | <0,0001 | <0,0001 |  |
| 0 dpi vs. 4 dpi | <0,0001 | <0,0001 | <0,0001 | <0,0001 |  |
| 0 dpi vs. 7 dpi | <0,0001 | <0,0001 | <0,0001 | <0,0001 |  |
| 0 dpi vs. 10 dpi | <0,0001 | <0,0001 | <0,0001 | <0,0001 |  |
| 0 dpi vs. 14 dpi | 0,9956 | <0,0001 | <0,0001 | <0,0001 |  |
| 0 dpi vs. 18 dpi | >0,9999 | <0,0001 | <0,0001 | <0,0001 |  |
| 0 dpi vs. 35 dpi | 0,9749 | <0,0001 | <0,0001 | <0,0001 |  |
| 0 dpi vs. 42 dpi | 0,9995 | <0,0001 | <0,0001 | <0,0001 |  |
| 0 dpi vs. 55 dpi | 0,9155 | <0,0001 | <0,0001 | <0,0001 |  |
| 0 dpi vs. 60 dpi | 0,0016 | <0,0001 | <0,0001 | <0,0001 |  |
| 2 dpi vs. 4 dpi | <0,0001 | >0,9999 | >0,9999 | >0,9999 |  |
| 2 dpi vs. 7 dpi | <0,0001 | <0,0001 | 0,9799 | >0,9999 |  |
| 2 dpi vs. 10 dpi | <0,0001 | <0,0001 | >0,9999 | >0,9999 |  |
| 2 dpi vs. 14 dpi | <0,0001 | <0,0001 | 0,0078 | >0,9999 |  |
| 2 dpi vs. 18 dpi | <0,0001 | <0,0001 | 0,0008 | >0,9999 |  |
| 2 dpi vs. 35 dpi | <0,0001 | <0,0001 | 0,0063 | 0,9972 |  |
| 2 dpi vs. 42 dpi | <0,0001 | <0,0001 | 0,0024 | 0,9996 |  |
| 2 dpi vs. 55 dpi | <0,0001 | <0,0001 | <0,0001 | 0,6213 |  |
| 2 dpi vs. 60 dpi | <0,0001 | <0,0001 | <0,0001 | 0,778 |  |
| 4 dpi vs. 7 dpi | <0,0001 | <0,0001 | 0,9799 | >0,9999 |  |
| 4 dpi vs. 10 dpi | <0,0001 | <0,0001 | >0,9999 | >0,9999 |  |
| 4 dpi vs. 14 dpi | <0,0001 | <0,0001 | 0,0078 | >0,9999 |  |
| 4 dpi vs. 18 dpi | <0,0001 | <0,0001 | 0,0008 | >0,9999 |  |
| 4 dpi vs. 35 dpi | <0,0001 | <0,0001 | 0,0063 | 0,9979 |  |
| 4 dpi vs. 42 dpi | <0,0001 | <0,0001 | 0,0024 | 0,9996 |  |
| 4 dpi vs. 55 dpi | <0,0001 | <0,0001 | <0,0001 | 0,6558 |  |
| 4 dpi vs. 60 dpi | <0,0001 | <0,0001 | <0,0001 | 0,8031 |  |
| 7 dpi vs. 10 dpi | <0,0001 | <0,0001 | 0,9964 | >0,9999 |  |
| 7 dpi vs. 14 dpi | <0,0001 | <0,0001 | 0,1027 | >0,9999 |  |
| 7 dpi vs. 18 dpi | <0,0001 | <0,0001 | 0,0126 | >0,9999 |  |
| 7 dpi vs. 35 dpi | <0,0001 | <0,0001 | 0,0859 | 0,9965 |  |
| 7 dpi vs. 42 dpi | <0,0001 | <0,0001 | 0,0367 | 0,9995 |  |
| 7 dpi vs. 55 dpi | <0,0001 | <0,0001 | <0,0001 | 0,5913 |  |
| 7 dpi vs. 60 dpi | <0,0001 | <0,0001 | <0,0001 | 0,7555 |  |
| 10 dpi vs. 14 dpi | <0,0001 | <0,0001 | 0,0181 | >0,9999 |  |
| 10 dpi vs. 18 dpi | <0,0001 | <0,0001 | 0,002 | >0,9999 |  |
| 10 dpi vs. 35 dpi | <0,0001 | <0,0001 | 0,0149 | 0,9979 |  |
| 10 dpi vs. 42 dpi | <0,0001 | <0,0001 | 0,0061 | 0,9996 |  |
| 10 dpi vs. 55 dpi | <0,0001 | <0,0001 | <0,0001 | 0,6558 |  |
| 10 dpi vs. 60 dpi | <0,0001 | <0,0001 | <0,0001 | 0,8031 |  |
| 14 dpi vs. 18 dpi | 0,9952 | <0,0001 | 0,9917 | >0,9999 |  |
| 14 dpi vs. 35 dpi | 0,4873 | <0,0001 | >0,9999 | 0,9972 |  |
| 14 dpi vs. 42 dpi | 0,8177 | <0,0001 | >0,9999 | 0,9996 |  |
| 14 dpi vs. 55 dpi | >0,9999 | >0,9999 | 0,0002 | 0,6213 |  |
| 14 dpi vs. 60 dpi | 0,0765 | 0,1442 | 0,2449 | 0,778 |  |
| 18 dpi vs. 35 dpi | 0,9972 | >0,9999 | 0,9947 | 0,9997 |  |
| 18 dpi vs. 42 dpi | >0,9999 | >0,9999 | 0,9998 | >0,9999 |  |
| 18 dpi vs. 55 dpi | 0,9332 | 0,0002 | 0,0959 | 0,852 |  |
| 18 dpi vs. 60 dpi | 0,0083 | <0,0001 | 0,9841 | 0,9281 |  |
| 35 dpi vs. 42 dpi | >0,9999 | >0,9999 | >0,9999 | >0,9999 |  |
| 35 dpi vs. 55 dpi | 0,1892 | <0,0001 | 0,0002 | 0,9775 |  |
| 35 dpi vs. 60 dpi | <0,0001 | <0,0001 | 0,2806 | 0,9958 |  |
| 42 dpi vs. 55 dpi | 0,4872 | <0,0001 | 0,002 | 0,9775 |  |
| 42 dpi vs. 60 dpi | <0,0001 | <0,0001 | 0,5729 | 0,9951 |  |
| 55 dpi vs. 60 dpi | 0,2604 | 0,1391 | 0,5689 | >0,9999 |  |
|  | **0 dpi** | **2 dpi** | **4 dpi** | **7 dpi** |  |
| 6w-old vs. 12w-old | <0,0001 | >0,9999 | <0,0001 | <0,0001 |  |
| 6w-old vs. 18w-old | <0,0001 | >0,9999 | <0,0001 | <0,0001 |  |
| 6w-old vs. 24w-old | <0,0001 | >0,9999 | <0,0001 | <0,0001 |  |
| 12w-old vs. 18w-old | <0,0001 | >0,9999 | >0,9999 | <0,0001 |  |
| 12w-old vs. 24w-old | <0,0001 | >0,9999 | >0,9999 | <0,0001 |  |
| 18w-old vs. 24w-old | <0,0001 | >0,9999 | >0,9999 | 0,4948 |  |
|  | **10 dpi** | **14 dpi** | **18 dpi** | **35 dpi** |  |
| 6w-old vs. 12w-old | <0,0001 | <0,0001 | <0,0001 | <0,0001 |  |
| 6w-old vs. 18w-old | <0,0001 | <0,0001 | <0,0001 | <0,0001 |  |
| 6w-old vs. 24w-old | <0,0001 | <0,0001 | <0,0001 | <0,0001 |  |
| 12w-old vs. 18w-old | <0,0001 | <0,0001 | <0,0001 | <0,0001 |  |
| 12w-old vs. 24w-old | <0,0001 | <0,0001 | <0,0001 | <0,0001 |  |
| 18w-old vs. 24w-old | 0,9956 | 0,0001 | 0,0006 | 0,0031 |  |
|  | **42 dpi** | **55 dpi** | **60 dpi** |  |  |
| 6w-old vs. 12w-old | <0,0001 | <0,0001 | <0,0001 |  |  |
| 6w-old vs. 18w-old | <0,0001 | <0,0001 | <0,0001 |  |  |
| 6w-old vs. 24w-old | <0,0001 | <0,0001 | <0,0001 |  |  |
| 12w-old vs. 18w-old | <0,0001 | <0,0001 | <0,0001 |  |  |
| 12w-old vs. 24w-old | <0,0001 | <0,0001 | <0,0001 |  |  |
| 18w-old vs. 24w-old | 0,0026 | <0,0001 | 0,0002 |  |  |
| **Figure 2b** |  |  |  |  |  |
| **Two-way ANOVA** | **6w-old** | **12w-old** | **18w-old** | **24w-old** |  |
| 0 dpi vs. 1 dpi | 0,0479 | 0,0077 | 0,0775 | 0,0241 |  |
| 0 dpi vs. 4 dpi | 0,0037 | 0,0026 | 0,0443 | 0,0047 |  |
| 0 dpi vs. 7 dpi | 0,0004 | 0,0008 | 0,0051 | 0,0011 |  |
| 0 dpi vs. 10 dpi | 0,0015 | <0,0001 | 0,0017 | <0,0001 |  |
| 0 dpi vs. 14 dpi | 0,0051 | <0,0001 | <0,0001 | <0,0001 |  |
| 0 dpi vs. 21 dpi | 0,2541 | 0,0001 | 0,0019 | <0,0001 |  |
| 0 dpi vs. 25 dpi | 0,8026 | 0,0003 | 0,0031 | <0,0001 |  |
| 0 dpi vs. 35 dpi | 0,9599 | 0,0013 | 0,0015 | <0,0001 |  |
| 0 dpi vs. 42 dpi | 0,9778 | 0,0057 | 0,0017 | 0,0003 |  |
| 0 dpi vs. 55 dpi | 0,9912 | 0,0015 | <0,0001 | 0,0044 |  |
| 0 dpi vs. 65 dpi | 0,982 | 0,0045 | <0,0001 | 0,0723 |  |
| 1 dpi vs. 4 dpi | 0,9996 | >0,9999 | >0,9999 | >0,9999 |  |
| 1 dpi vs. 7 dpi | 0,9527 | >0,9999 | 0,9964 | 0,9995 |  |
| 1 dpi vs. 10 dpi | 0,9954 | 0,7216 | 0,9627 | 0,7354 |  |
| 1 dpi vs. 14 dpi | 0,9999 | 0,6722 | 0,1987 | 0,6887 |  |
| 1 dpi vs. 21 dpi | 0,9999 | 0,9891 | 0,9685 | 0,4569 |  |
| 1 dpi vs. 25 dpi | 0,8866 | 0,9965 | 0,9873 | 0,8017 |  |
| 1 dpi vs. 35 dpi | 0,9124 | >0,9999 | 0,9549 | 0,5988 |  |
| 1 dpi vs. 42 dpi | 0,8646 | >0,9999 | 0,9616 | 0,9425 |  |
| 1 dpi vs. 55 dpi | 0,7827 | >0,9999 | 0,2839 | 0,9716 |  |
| 1 dpi vs. 65 dpi | 0,8463 | >0,9999 | 0,4442 | 0,9674 |  |
| 4 dpi vs. 7 dpi | >0,9999 | >0,9999 | 0,9997 | >0,9999 |  |
| 4 dpi vs. 10 dpi | >0,9999 | 0,9492 | 0,9916 | 0,9281 |  |
| 4 dpi vs. 14 dpi | >0,9999 | 0,93 | 0,3286 | 0,9034 |  |
| 4 dpi vs. 21 dpi | 0,9231 | 0,9999 | 0,9934 | 0,7223 |  |
| 4 dpi vs. 25 dpi | 0,3543 | >0,9999 | 0,9981 | 0,9549 |  |
| 4 dpi vs. 35 dpi | 0,4875 | >0,9999 | 0,9891 | 0,8354 |  |
| 4 dpi vs. 42 dpi | 0,4056 | >0,9999 | 0,9913 | 0,9937 |  |
| 4 dpi vs. 55 dpi | 0,3073 | >0,9999 | 0,4341 | 0,9964 |  |
| 4 dpi vs. 65 dpi | 0,38 | >0,9999 | 0,6085 | 0,9901 |  |
| 7 dpi vs. 10 dpi | >0,9999 | 0,9795 | >0,9999 | 0,9934 |  |
| 7 dpi vs. 14 dpi | >0,9999 | 0,9689 | 0,8575 | 0,989 |  |
| 7 dpi vs. 21 dpi | 0,5674 | >0,9999 | >0,9999 | 0,9243 |  |
| 7 dpi vs. 25 dpi | 0,0858 | >0,9999 | >0,9999 | 0,9968 |  |
| 7 dpi vs. 35 dpi | 0,1798 | >0,9999 | >0,9999 | 0,9673 |  |
| 7 dpi vs. 42 dpi | 0,1355 | >0,9999 | >0,9999 | 0,9998 |  |
| 7 dpi vs. 55 dpi | 0,0905 | >0,9999 | 0,9139 | 0,9998 |  |
| 7 dpi vs. 65 dpi | 0,123 | >0,9999 | 0,9686 | 0,9978 |  |
| 10 dpi vs. 14 dpi | >0,9999 | >0,9999 | 0,9688 | >0,9999 |  |
| 10 dpi vs. 21 dpi | 0,8136 | 0,9998 | >0,9999 | >0,9999 |  |
| 10 dpi vs. 25 dpi | 0,215 | 0,9995 | >0,9999 | >0,9999 |  |
| 10 dpi vs. 35 dpi | 0,3444 | 0,9834 | >0,9999 | >0,9999 |  |
| 10 dpi vs. 42 dpi | 0,2749 | 0,8655 | >0,9999 | >0,9999 |  |
| 10 dpi vs. 55 dpi | 0,1975 | 0,9934 | 0,9852 | >0,9999 |  |
| 10 dpi vs. 65 dpi | 0,2542 | 0,9526 | 0,9967 | >0,9999 |  |
| 14 dpi vs. 21 dpi | 0,9487 | 0,9995 | 0,963 | >0,9999 |  |
| 14 dpi vs. 25 dpi | 0,414 | 0,9988 | 0,9248 | >0,9999 |  |
| 14 dpi vs. 35 dpi | 0,5427 | 0,9746 | 0,9747 | >0,9999 |  |
| 14 dpi vs. 42 dpi | 0,4584 | 0,8306 | 0,9697 | >0,9999 |  |
| 14 dpi vs. 55 dpi | 0,3542 | 0,9893 | >0,9999 | >0,9999 |  |
| 14 dpi vs. 65 dpi | 0,4317 | 0,935 | >0,9999 | >0,9999 |  |
| 21 dpi vs. 25 dpi | 0,9988 | >0,9999 | >0,9999 | >0,9999 |  |
| 21 dpi vs. 35 dpi | 0,9982 | >0,9999 | >0,9999 | >0,9999 |  |
| 21 dpi vs. 42 dpi | 0,9949 | 0,9983 | >0,9999 | >0,9999 |  |
| 21 dpi vs. 55 dpi | 0,9848 | >0,9999 | 0,982 | >0,9999 |  |
| 21 dpi vs. 60 dpi | 0,9932 | 0,9999 | 0,9958 | >0,9999 |  |
| 25 dpi vs. 35 dpi | >0,9999 | >0,9999 | >0,9999 | >0,9999 |  |
| 25 dpi vs. 42 dpi | >0,9999 | 0,9996 | >0,9999 | >0,9999 |  |
| 25 dpi vs. 55 dpi | >0,9999 | >0,9999 | 0,9592 | >0,9999 |  |
| 25 dpi vs. 65 dpi | >0,9999 | >0,9999 | 0,9879 | >0,9999 |  |
| 35 dpi vs. 42 dpi | >0,9999 | >0,9999 | >0,9999 | >0,9999 |  |
| 35 dpi vs. 55 dpi | >0,9999 | >0,9999 | 0,9883 | >0,9999 |  |
| 35 dpi vs. 65 dpi | >0,9999 | >0,9999 | 0,9976 | >0,9999 |  |
| 42 dpi vs. 55 dpi | >0,9999 | >0,9999 | 0,9857 | >0,9999 |  |
| 42 dpi vs. 65 dpi | >0,9999 | >0,9999 | 0,9968 | >0,9999 |  |
| 55 dpi vs. 65 dpi | >0,9999 | >0,9999 | >0,9999 | >0,9999 |  |
|  | **0 dpi** | **1 dpi** | **4 dpi** | **7 dpi** |  |
| 6w-old vs. 12w-old | >0,9999 | 0,8166 | 0,9627 | 0,9997 |  |
| 6w-old vs. 18w-old | 0,9384 | 0,6899 | 0,9552 | 0,8974 |  |
| 6w-old vs. 24w-old | 0,9995 | 0,9031 | 0,9873 | 0,9992 |  |
| 12w-old vs. 18w-old | 0,9477 | 0,9961 | >0,9999 | 0,93 |  |
| 12w-old vs. 24w-old | 0,9997 | 0,9983 | 0,9985 | >0,9999 |  |
| 18w-old vs. 24w-old | 0,9678 | 0,9809 | 0,9978 | 0,9407 |  |
|  | **10 dpi** | **14 dpi** | **21 dpi** | **25 dpi** |  |
| 6w-old vs. 12w-old | 0,2962 | 0,1337 | 0,0276 | 0,0029 |  |
| 6w-old vs. 18w-old | 0,4859 | 0,0102 | 0,0083 | 0,0005 |  |
| 6w-old vs. 24w-old | 0,3669 | 0,1769 | 0,001 | 0,0002 |  |
| 12w-old vs. 18w-old | 0,9871 | 0,7718 | 0,9788 | 0,9851 |  |
| 12w-old vs. 24w-old | 0,9991 | 0,9991 | 0,7068 | 0,9159 |  |
| 18w-old vs. 24w-old | 0,9971 | 0,6928 | 0,9019 | 0,989 |  |
|  | **35 dpi** | **42 dpi** | **55 dpi** | **65 dpi** |  |
| 6w-old vs. 12w-old | 0,0258 | 0,0529 | 0,0118 | 0,0369 |  |
| 6w-old vs. 18w-old | 0,0013 | 0,0009 | <0,0001 | <0,0001 |  |
| 6w-old vs. 24w-old | 0,0005 | 0,0036 | 0,0103 | 0,0583 |  |
| 12w-old vs. 18w-old | 0,7655 | 0,5095 | 0,2071 | 0,1719 |  |
| 12w-old vs. 24w-old | 0,5151 | 0,65 | 0,9005 | 0,6846 |  |
| 18w-old vs. 24w-old | 0,9643 | 0,9998 | 0,8586 | 0,9998 |  |
| **Figure 3a** |  |  |  |  |  |
| **Two-way ANOVA** | **6w-old** | **12w-old** | **18w-old** | **24w-old** |  |
| 0 dpi vs. 3 dpi | <0,0001 | <0,0001 | <0,0001 | <0,0001 |  |
| 0 dpi vs. 7 dpi | 0,9132 | 0,0272 | <0,0001 | <0,0001 |  |
| 0 dpi vs. 14 dpi | 0,9839 | 0,9997 | >0,9999 | 0,9933 |  |
| 3 dpi vs. 7 dpi | <0,0001 | <0,0001 | 0,0056 | 0,0008 |  |
| 3 dpi vs. 14 dpi | <0,0001 | <0,0001 | <0,0001 | <0,0001 |  |
| 7 dpi vs. 14 dpi | 0,7428 | 0,0218 | <0,0001 | <0,0001 |  |
|  | **0 dpi** | **3 dpi** | **7 dpi** | **14 dpi** |  |
| 6w-old vs. 12w-old | 0,9981 | 0,0007 | 0,1616 | 0,9997 |  |
| 6w-old vs. 18w-old | 0,9628 | <0,0001 | 0,0003 | 0,9999 |  |
| 6w-old vs. 24w-old | 0,9288 | <0,0001 | <0,0001 | >0,9999 |  |
| 12w-old vs. 18w-old | 0,9898 | <0,0001 | 0,0769 | 0,9985 |  |
| 12w-old vs. 24w-old | 0,9721 | 0,0001 | 0,0003 | 0,9998 |  |
| 18w-old vs. 24w-old | 0,9992 | 0,0363 | 0,1598 | 0,9998 |  |
| **Figure 3b** |  |  |  |  |  |
| **Two-way ANOVA** | **6w-old** | **12w-old** | **18w-old** | **24w-old** |  |
| 0 dpi vs. 3 dpi | <0,0001 | 0,0023 | 0,0036 | 0,3134 |  |
| 0 dpi vs. 7 dpi | 0,2394 | 0,1301 | 0,0088 | 0,447 |  |
| 0 dpi vs. 14 dpi | 0,7949 | 0,9983 | 0,9582 | 0,9944 |  |
| 3 dpi vs. 7 dpi | 0,0029 | 0,3552 | 0,9869 | 0,9944 |  |
| 3 dpi vs. 14 dpi | 0,0002 | 0,0015 | 0,0134 | 0,447 |  |
| 7 dpi vs. 14 dpi | 0,749 | 0,0929 | 0,0304 | 0,5985 |  |
|  | **0 dpi** | **3 dpi** | **7 dpi** | **14 dpi** |  |
| 6w-old vs. 12w-old | 0,9998 | 0,3134 | 0,975 | 0,749 |  |
| 6w-old vs. 18w-old | 0,4963 | 0,0088 | 0,9998 | 0,2747 |  |
| 6w-old vs. 24w-old | 0,9082 | 0,0003 | 0,7003 | 0,547 |  |
| 12w-old vs. 18w-old | 0,447 | 0,3552 | 0,9869 | 0,8372 |  |
| 12w-old vs. 24w-old | 0,8752 | 0,0304 | 0,447 | 0,9869 |  |
| 18w-old vs. 24w-old | 0,8752 | 0,5985 | 0,6499 | 0,9582 |  |
| **Figure 3c** |  |  |  |  |  |
| **Two-way ANOVA** | **6w-old** | **12w-old** | **18w-old** | **24w-old** |  |
| 0 dpi vs. 2 dpi | 0,9997 | 0,9997 | 0,9729 | 0,9811 |  |
| 0 dpi vs. 7 dpi | 0,0843 | <0,0001 | <0,0001 | <0,0001 |  |
| 0 dpi vs. 14 dpi | 0,9882 | 0,3705 | 0,0394 | 0,2698 |  |
| 0 dpi vs. 21 dpi | 0,9996 | >0,9999 | 0,953 | 0,9998 |  |
| 2 dpi vs. 7 dpi | 0,1896 | <0,0001 | <0,0001 | <0,0001 |  |
| 2 dpi vs. 14 dpi | 0,9987 | 0,4908 | 0,2091 | 0,0988 |  |
| 2 dpi vs. 21 dpi | 0,9976 | 0,9983 | >0,9999 | 0,9593 |  |
| 7 dpi vs. 14 dpi | 0,3171 | <0,0001 | <0,0001 | <0,0001 |  |
| 7 dpi vs. 21 dpi | 0,3579 | <0,0001 | <0,0001 | <0,0001 |  |
| 14 dpi vs. 21 dpi | 0,9854 | 0,2367 | 0,1726 | 0,4233 |  |
|  | **0 dpi** | **2 dpi** | **7 dpi** | **14 dpi** | **21 dpi** |
| 6w-old vs. 12w-old | 0,9997 | 0,9997 | <0,0001 | 0,4943 | 0,9971 |
| 6w-old vs. 18w-old | 0,9953 | 0,9978 | <0,0001 | 0,1751 | 0,9588 |
| 6w-old vs. 24w-old | 0,9453 | 0,9973 | <0,0001 | 0,2345 | 0,9167 |
| 12w-old vs. 18w-old | 0,9888 | 0,9997 | 0,0808 | 0,8034 | 0,9644 |
| 12w-old vs. 24w-old | 0,9757 | 0,9912 | 0,1137 | 0,8869 | 0,9032 |
| 18w-old vs. 24w-old | 0,8586 | 0,9796 | 0,9986 | 0,9985 | 0,9948 |
| **Figure 3e** |  |  |  |  |  |
| **Two-way ANOVA** | **6w-old** | **12w-old** | **18w-old** | **24w-old** |  |
| 0 dpi vs. 2 dpi | 0,9364 | 0,9866 | 0,9997 | 0,7992 |  |
| 0 dpi vs. 7 dpi | <0,0001 | <0,0001 | <0,0001 | 0,7721 |  |
| 0 dpi vs. 14 dpi | <0,0001 | <0,0001 | <0,0001 | <0,0001 |  |
| 0 dpi vs. 21 dpi | <0,0001 | <0,0001 | <0,0001 | <0,0001 |  |
| 2 dpi vs. 7 dpi | 0,0001 | <0,0001 | <0,0001 | 0,2138 |  |
| 2 dpi vs. 14 dpi | 0,001 | <0,0001 | <0,0001 | <0,0001 |  |
| 2 dpi vs. 21 dpi | <0,0001 | <0,0001 | <0,0001 | <0,0001 |  |
| 7 dpi vs. 14 dpi | 0,9972 | 0,8468 | 0,0585 | 0,0003 |  |
| 7 dpi vs. 21 dpi | 0,0585 | 0,1871 | 0,0002 | <0,0001 |  |
| 14 dpi vs. 21 dpi | 0,0306 | 0,7401 | 0,5861 | 0,0048 |  |
|  | **0 dpi** | **2 dpi** | **7 dpi** | **14 dpi** | **21 dpi** |
| 6w-old vs. 12w-old | 0,4801 | 0,0908 | 0,9982 | 0,6413 | 0,9636 |
| 6w-old vs. 18w-old | 0,0148 | 0,0022 | 0,1516 | 0,7784 | 0,9329 |
| 6w-old vs. 24w-old | 0,6583 | 0,0289 | <0,0001 | >0,9999 | 0,9185 |
| 12w-old vs. 18w-old | 0,3774 | 0,7414 | 0,2463 | 0,9957 | 0,9998 |
| 12w-old vs. 24w-old | 0,9971 | 0,9744 | 0,0001 | 0,6664 | 0,6933 |
| 18w-old vs. 24w-old | 0,3187 | 0,9431 | 0,0261 | 0,8002 | 0,5907 |
| **Figure 3f** |  |  |  |  |  |
| **Two-way ANOVA Bioc^-^/DAPI^+^** | **6w-old** | **12w-old** | **18w-old** | **24w-old** |  |
| 0 dpi vs. 2 dpi | <0,0001 | <0,0001 | <0,0001 | <0,0001 |  |
| 0 dpi vs. 7 dpi | >0,9999 | 0,9476 | 0,357 | 0,0031 |  |
| 0 dpi vs. 14 dpi | 0,9995 | 0,756 | 0,9615 | 0,9972 |  |
| 0 dpi vs. 21 dpi | 0,3085 | 0,045 | 0,0002 | 0,0824 |  |
| 2 dpi vs. 7 dpi | <0,0001 | <0,0001 | <0,0001 | <0,0001 |  |
| 2 dpi vs. 14 dpi | <0,0001 | <0,0001 | <0,0001 | <0,0001 |  |
| 2 dpi vs. 21 dpi | <0,0001 | <0,0001 | <0,0001 | <0,0001 |  |
| 7 dpi vs. 14 dpi | >0,9999 | 0,391 | 0,1406 | 0,0015 |  |
| 7 dpi vs. 21 dpi | 0,4331 | 0,0123 | <0,0001 | <0,0001 |  |
| 14 dpi vs. 21 dpi | 0,5141 | 0,533 | 0,0069 | 0,2011 |  |
|  | **0 dpi** | **2 dpi** | **7 dpi** | **14 dpi** | **21 dpi** |
| 6w-old vs. 12w-old | 0,4426 | 0,037 | 0,1517 | 0,9851 | 0,9901 |
| 6w-old vs. 18w-old | 0,3709 | 0,0002 | 0,0055 | 0,788 | 0,8317 |
| 6w-old vs. 24w-old | 0,3343 | 0,0083 | <0,0001 | 0,5199 | 0,86 |
| 12w-old vs. 18w-old | 0,9993 | 0,624 | 0,7116 | 0,9379 | 0,674 |
| 12w-old vs. 24w-old | 0,9926 | 0,963 | 0,0108 | 0,7384 | 0,9692 |
| 18w-old vs. 24w-old | 0,9985 | 0,9003 | 0,1016 | 0,9716 | 0,3486 |
| **Two-way ANOVA Bioc^+^/DAPI^+^** | **6w-old** | **12w-old** | **18w-old** | **24w-old** |  |
| 0 dpi vs. 2 dpi | <0,0001 | <0,0001 | <0,0001 | <0,0001 |  |
| 0 dpi vs. 7 dpi | <0,0001 | <0,0001 | <0,0001 | <0,0001 |  |
| 0 dpi vs. 14 dpi | <0,0001 | <0,0001 | <0,0001 | <0,0001 |  |
| 0 dpi vs. 21 dpi | <0,0001 | <0,0001 | <0,0001 | <0,0001 |  |
| 2 dpi vs. 7 dpi | <0,0001 | 0,0455 | <0,0001 | 0,0004 |  |
| 2 dpi vs. 14 dpi | <0,0001 | 0,0013 | 0,0007 | 0,0006 |  |
| 2 dpi vs. 21 dpi | 0,0014 | 0,0003 | <0,0001 | 0,1123 |  |
| 7 dpi vs. 14 dpi | 0,998 | 0,9233 | 0,9999 | >0,9999 |  |
| 7 dpi vs. 21 dpi | 0,8932 | 0,6808 | 0,1174 | 0,4941 |  |
| 14 dpi vs. 21 dpi | 0,6196 | 0,9983 | 0,078 | 0,6293 |  |
|  | **0 dpi** | **2 dpi** | **7 dpi** | **14 dpi** | **21 dpi** |
| 6w-old vs. 12w-old | 0,998 | 0,915 | 0,0378 | 0,1796 | 0,9968 |
| 6w-old vs. 18w-old | 0,2936 | 0,7141 | 0,2538 | 0,0585 | 0,2101 |
| 6w-old vs. 24w-old | 0,8479 | 0,8691 | 0,6782 | 0,214 | 0,2199 |
| 12w-old vs. 18w-old | 0,2139 | 0,9875 | 0,7562 | 0,9594 | 0,3501 |
| 12w-old vs. 24w-old | 0,9169 | 0,9995 | 0,4858 | 0,9997 | 0,1698 |
| 18w-old vs. 24w-old | 0,0668 | 0,9965 | 0,9441 | 0,9354 | 0,0007 |
| **Figure 3h** |  |  |  |  |  |
| **Two-way ANOVA** | **6w-old** | **12w-old** | **18w-old** | **24w-old** |  |
| 0 dpi vs. 2 dpi | <0,0001 | <0,0001 | <0,0001 | <0,0001 |  |
| 0 dpi vs. 3 dpi | <0,0001 | <0,0001 | <0,0001 | <0,0001 |  |
| 0 dpi vs. 4 dpi | <0,0001 | <0,0001 | <0,0001 | <0,0001 |  |
| 0 dpi vs. 7 dpi | <0,0001 | <0,0001 | <0,0001 | <0,0001 |  |
| 0 dpi vs. 14 dpi | <0,0001 | <0,0001 | <0,0001 | <0,0001 |  |
| 0 dpi vs. 21 dpi | <0,0001 | <0,0001 | <0,0001 | <0,0001 |  |
| 2 dpi vs. 3 dpi | 0,1923 | 0,998 | 0,9997 | 0,9995 |  |
| 2 dpi vs. 4 dpi | <0,0001 | 0,1219 | 0,218 | 0,1816 |  |
| 2 dpi vs. 7 dpi | <0,0001 | 0,0387 | 0,0004 | 0,0001 |  |
| 2 dpi vs. 14 dpi | <0,0001 | 0,0009 | 0,0035 | 0,0002 |  |
| 2 dpi vs. 21 dpi | 0,0012 | 0,0002 | <0,0001 | 0,0711 |  |
| 3 dpi vs. 4 dpi | 0,0781 | 0,317 | 0,4747 | 0,3555 |  |
| 3 dpi vs. 7 dpi | 0,0401 | 0,1243 | 0,0035 | 0,0003 |  |
| 3 dpi vs. 14 dpi | 0,0099 | 0,0042 | 0,0192 | 0,0005 |  |
| 3 dpi vs. 21 dpi | 0,586 | 0,001 | <0,0001 | 0,1607 |  |
| 4 dpi vs. 7 dpi | >0,9999 | 0,9993 | 0,5615 | 0,2022 |  |
| 4 dpi vs. 14 dpi | 0,9938 | 0,6884 | 0,8039 | 0,2972 |  |
| 4 dpi vs. 21 dpi | 0,9454 | 0,3743 | 0,0019 | 0,9996 |  |
| 7 dpi vs. 14 dpi | 0,9979 | 0,9165 | >0,9999 | >0,9999 |  |
| 7 dpi vs. 21 dpi | 0,8893 | 0,6611 | 0,208 | 0,411 |  |
| 14 dpi vs. 21 dpi | 0,6103 | 0,9981 | 0,1512 | 0,5523 |  |
|  | **0 dpi** | **2 dpi** | **3 dpi** | **4 dpi** |  |
| 6w-old vs. 12w-old | >0,9999 | 0,9163 | 0,0746 | 0,021 |  |
| 6w-old vs. 18w-old | >0,9999 | 0,6727 | 0,0142 | 0,0013 |  |
| 6w-old vs. 24w-old | >0,9999 | 0,8851 | 0,0443 | 0,0094 |  |
| 12w-old vs. 18w-old | >0,9999 | 0,9787 | 0,9345 | 0,8308 |  |
| 12w-old vs. 24w-old | >0,9999 | 0,9998 | 0,9971 | 0,9933 |  |
| 18w-old vs. 24w-old | >0,9999 | 0,9892 | 0,9796 | 0,9375 |  |
|  | **7 dpi** | **14 dpi** | **21 dpi** |  |  |
| 6w-old vs. 12w-old | 0,0401 | 0,1936 | 0,9932 |  |  |
| 6w-old vs. 18w-old | 0,0736 | 0,0143 | 0,6485 |  |  |
| 6w-old vs. 24w-old | 0,8528 | 0,3551 | 0,3095 |  |  |
| 12w-old vs. 18w-old | 0,9785 | 0,7233 | 0,8346 |  |  |
| 12w-old vs. 24w-old | 0,3211 | 0,9854 | 0,2176 |  |  |
| 18w-old vs. 24w-old | 0,4854 | 0,5053 | 0,0183 |  |  |
| **Figure 4b** |  |  |  |  |  |
| **Two-way ANOVA** | **6w-old** | **12w-old** | **18w-old** | **24w-old** |  |
| 0 dpi vs. 7 dpi | 0,0026 | <0,0001 | <0,0001 | <0,0001 |  |
| 0 dpi vs. 14 dpi | 0,7233 | 0,0002 | <0,0001 | <0,0001 |  |
| 0 dpi vs. 21 dpi | 0,3216 | 0,6329 | <0,0001 | <0,0001 |  |
| 7 dpi vs. 14 dpi | 0,1055 | <0,0001 | 0,0316 | 0,2135 |  |
| 7 dpi vs. 21 dpi | 0,2318 | <0,0001 | <0,0001 | 0,0355 |  |
| 14 dpi vs. 21 dpi | 0,9464 | 0,0104 | 0,0935 | 0,8388 |  |
|  | **0 dpi** | **7 dpi** | **14 dpi** | **21 dpi** |  |
| 6w-old vs. 12w-old | >0,9999 | <0,0001 | 0,0126 | 0,9637 |  |
| 6w-old vs. 18w-old | >0,9999 | <0,0001 | <0,0001 | 0,0039 |  |
| 6w-old vs. 24w-old | >0,9999 | <0,0001 | <0,0001 | <0,0001 |  |
| 12w-old vs. 18w-old | >0,9999 | 0,9987 | 0,0299 | 0,0012 |  |
| 12w-old vs. 24w-old | >0,9999 | 0,9973 | 0,0007 | <0,0001 |  |
| 18w-old vs. 24w-old | >0,9999 | >0,9999 | 0,8227 | 0,0393 |  |
| **Figure 4c** |  |  |  |  |  |
| **Two-way ANOVA** | **6w-old** | **12w-old** | **18w-old** | **24w-old** |  |
| 0 dpi vs. 2 dpi | 0,0004 | 0,3159 | 0,1853 | 0,9985 |  |
| 0 dpi vs. 7 dpi | <0,0001 | <0,0001 | 0,0005 | 0,0136 |  |
| 0 dpi vs. 14 dpi | 0,0057 | <0,0001 | <0,0001 | 0,008 |  |
| 0 dpi vs. 21 dpi | 0,2738 | <0,0001 | <0,0001 | 0,1167 |  |
| 2 dpi vs. 7 dpi | 0,9957 | 0,0067 | 0,3677 | 0,0194 |  |
| 2 dpi vs. 14 dpi | 0,937 | <0,0001 | 0,0085 | 0,0123 |  |
| 2 dpi vs. 21 dpi | 0,1022 | 0,0041 | 0,075 | 0,1175 |  |
| 7 dpi vs. 14 dpi | 0,7506 | 0,4213 | 0,396 | 0,9998 |  |
| 7 dpi vs. 21 dpi | 0,0231 | 0,9998 | 0,9007 | 0,9798 |  |
| 14 dpi vs. 21 dpi | 0,4509 | 0,5259 | 0,8987 | 0,9495 |  |
|  | **0 dpi** | **2 dpi** | **7 dpi** | **14 dpi** | **21 dpi** |
| 6w-old vs. 12w-old | 0,9809 | 0,0683 | 0,9508 | 0,0089 | 0,0019 |
| 6w-old vs. 18w-old | 0,2698 | 0,9753 | 0,6973 | 0,0017 | <0,0001 |
| 6w-old vs. 24w-old | 0,0021 | 0,5513 | 0,4427 | 0,0384 | 0,0026 |
| 12w-old vs. 18w-old | 0,166 | 0,1662 | 0,9442 | 0,9314 | 0,5836 |
| 12w-old vs. 24w-old | 0,0012 | 0,635 | 0,7668 | 0,9351 | 0,998 |
| 18w-old vs. 24w-old | 0,3124 | 0,801 | 0,976 | 0,6361 | 0,7492 |
| **Figure 5a** |  |  |  |  |  |
| **Two-way ANOVA** | **6w-old** | **12w-old** | **18w-old** | **24w-old** |  |
| 0 dpi vs. 2 dpi | 0,0254 | 0,3016 | 0,2915 | 0,0069 |  |
| 0 dpi vs. 7 dpi | 0,3527 | 0,7176 | 0,0081 | 0,4154 |  |
| 0 dpi vs. 14 dpi | 0,962 | 0,6251 | 0,7832 | 0,9558 |  |
| 0 dpi vs. 21 dpi | 0,778 | 0,4327 | 0,7203 | 0,9977 |  |
| 2 dpi vs. 7 dpi | 0,7729 | 0,964 | 0,607 | 0,4388 |  |
| 2 dpi vs. 14 dpi | 0,1643 | 0,9959 | 0,9675 | 0,1187 |  |
| 2 dpi vs. 21 dpi | 0,5013 | >0,9999 | 0,9832 | 0,0515 |  |
| 7 dpi vs. 14 dpi | 0,7928 | 0,9992 | 0,2929 | 0,9088 |  |
| 7 dpi vs. 21 dpi | 0,9841 | 0,9832 | 0,3473 | 0,7362 |  |
| 14 dpi vs. 21 dpi | 0,9857 | 0,9987 | >0,9999 | 0,9971 |  |
|  | **0 dpi** | **2 dpi** | **7 dpi** | **14 dpi** | **21 dpi** |
| 6w-old vs. 12w-old | 0,6581 | >0,9999 | 0,9665 | 0,3265 | 0,506 |
| 6w-old vs. 18w-old | 0,4846 | 0,9955 | 0,0316 | 0,3381 | 0,6293 |
| 6w-old vs. 24w-old | 0,0064 | 0,005 | 0,0234 | 0,0261 | 0,24 |
| 12w-old vs. 18w-old | 0,9923 | 0,9931 | 0,0955 | >0,9999 | 0,9972 |
| 12w-old vs. 24w-old | 0,1202 | 0,0045 | 0,0736 | 0,6817 | 0,9585 |
| 18w-old vs. 24w-old | 0,2078 | 0,0099 | 0,9994 | 0,6687 | 0,8971 |
| **Figure 5b** |  |  |  |  |  |
| **Two-way ANOVA** | **6w-old** | **12w-old** | **18w-old** | **24w-old** |  |
| 0 dpi vs. 2 dpi | <0,0001 | 0,0098 | 0,0693 | 0,2598 |  |
| 0 dpi vs. 7 dpi | 0,0392 | 0,0112 | 0,0074 | 0,2332 |  |
| 0 dpi vs. 14 dpi | 0,9815 | 0,7686 | 0,9913 | 0,7139 |  |
| 0 dpi vs. 21 dpi | 0,6112 | 0,4821 | 0,9997 | 0,6615 |  |
| 2 dpi vs. 7 dpi | 0,0092 | 0,9985 | 0,9632 | >0,9999 |  |
| 2 dpi vs. 14 dpi | <0,0001 | 0,1038 | 0,1244 | 0,8867 |  |
| 2 dpi vs. 21 dpi | <0,0001 | 0,2595 | 0,0277 | 0,0113 |  |
| 7 dpi vs. 14 dpi | 0,1493 | 0,1297 | 0,0132 | 0,8916 |  |
| 7 dpi vs. 21 dpi | 0,4934 | 0,3282 | 0,0017 | 0,0072 |  |
| 14 dpi vs. 21 dpi | 0,9158 | 0,9866 | 0,9585 | 0,0669 |  |
|  | **0 dpi** | **2 dpi** | **7 dpi** | **14 dpi** | **21 dpi** |
| 6w-old vs. 12w-old | 0,9987 | 0,0931 | 0,9018 | 0,8781 | 0,9782 |
| 6w-old vs. 18w-old | 0,133 | 0,7059 | 0,0117 | 0,1291 | 0,8483 |
| 6w-old vs. 24w-old | 0,0348 | 0,6717 | 0,0998 | 0,0031 | 0,9993 |
| 12w-old vs. 18w-old | 0,1797 | 0,561 | 0,0666 | 0,3859 | 0,976 |
| 12w-old vs. 24w-old | 0,0506 | 0,5964 | 0,3518 | 0,0136 | 0,9948 |
| 18w-old vs. 24w-old | 0,9346 | >0,9999 | 0,8211 | 0,4095 | 0,923 |
| **Figure 6a** |  |  |  |  |  |
| **Two-way ANOVA** | **6w-old** | **12w-old** | **18w-old** | **24w-old** |  |
| 0 dpi vs. 2 dpi | 0,9896 | 0,9998 | 0,353 | 0,8227 |  |
| 0 dpi vs. 7 dpi | 0,9996 | 0,9679 | 0,924 | >0,9999 |  |
| 0 dpi vs. 14 dpi | >0,9999 | 0,9507 | 0,8452 | >0,9999 |  |
| 0 dpi vs. 21 dpi | 0,3292 | 0,2243 | 0,991 | 0,9905 |  |
| 2 dpi vs. 7 dpi | 0,965 | 0,9926 | 0,8059 | 0,7725 |  |
| 2 dpi vs. 14 dpi | 0,9899 | 0,9208 | 0,8907 | 0,8755 |  |
| 2 dpi vs. 21 dpi | 0,184 | 0,2233 | 0,6073 | 0,9793 |  |
| 7 dpi vs. 14 dpi | 0,9999 | 0,6552 | 0,9996 | 0,9999 |  |
| 7 dpi vs. 21 dpi | 0,4404 | 0,0576 | 0,9961 | 0,9806 |  |
| 14 dpi vs. 21 dpi | 0,4235 | 0,627 | 0,9794 | 0,9952 |  |
|  | **0 dpi** | **2 dpi** | **7 dpi** | **14 dpi** | **21 dpi** |
| 6w-old vs. 12w-old | 0,5616 | 0,49 | 0,965 | 0,2616 | 0,4251 |
| 6w-old vs. 18w-old | 0,5017 | 0,0069 | 0,1917 | 0,1319 | >0,9999 |
| 6w-old vs. 24w-old | 0,0823 | 0,0044 | 0,1551 | 0,1566 | 0,7744 |
| 12w-old vs. 18w-old | 0,9997 | 0,212 | 0,4134 | 0,9788 | 0,401 |
| 12w-old vs. 24w-old | 0,6736 | 0,1585 | 0,3525 | 0,9735 | 0,9667 |
| 18w-old vs. 24w-old | 0,7316 | 0,9987 | 0,9995 | >0,9999 | 0,7525 |
| **Figure 6b** |  |  |  |  |  |
| **Two-way ANOVA** | **6w-old** | **12w-old** | **18w-old** | **24w-old** |  |
| 0 dpi vs. 2 dpi | 0,2202 | 0,9536 | 0,555 | 0,98 |  |
| 0 dpi vs. 7 dpi | 0,0004 | 0,0175 | 0,0356 | 0,9676 |  |
| 0 dpi vs. 14 dpi | 0,095 | 0,0723 | 0,0656 | 0,1409 |  |
| 0 dpi vs. 21 dpi | 0,9036 | 0,0418 | 0,0146 | 0,439 |  |
| 2 dpi vs. 7 dpi | 0,2905 | 0,0045 | 0,7395 | >0,9999 |  |
| 2 dpi vs. 14 dpi | 0,9951 | 0,021 | 0,8582 | 0,4863 |  |
| 2 dpi vs. 21 dpi | 0,678 | 0,0111 | 0,5479 | 0,8206 |  |
| 7 dpi vs. 14 dpi | 0,5257 | 0,9977 | 0,9991 | 0,4278 |  |
| 7 dpi vs. 21 dpi | 0,0067 | 0,9973 | 0,9972 | 0,799 |  |
| 14 dpi vs. 21 dpi | 0,4211 | >0,9999 | 0,9771 | 0,9885 |  |
|  | **0 dpi** | **2 dpi** | **7 dpi** | **14 dpi** | **21 dpi** |
| 6w-old vs. 12w-old | 0,4295 | 0,5421 | 0,9896 | 0,4844 | 0,0045 |
| 6w-old vs. 18w-old | 0,1168 | 0,5092 | 0,8586 | 0,1772 | 0,0001 |
| 6w-old vs. 24w-old | 0,0444 | 0,8222 | 0,6895 | 0,1534 | 0,007 |
| 12w-old vs. 18w-old | 0,8764 | 0,0411 | 0,9631 | 0,9503 | 0,6625 |
| 12w-old vs. 24w-old | 0,6448 | 0,1348 | 0,4975 | 0,9288 | 0,9995 |
| 18w-old vs. 24w-old | 0,9738 | 0,9522 | 0,2435 | 0,9998 | 0,7787 |
| **Figure 7b** |  |  |  |  |  |
| **One-way ANOVA** | **7 dpi** |  |  |  |  |
| 6w-old vs. 12w-old | 0,1209 |  |  |  |  |
| 6w-old vs. 18w-old | 0,0002 |  |  |  |  |
| 6w-old vs. 24w-old | <0,0001 |  |  |  |  |
| 12w-old vs. 18w-old | 0,0977 |  |  |  |  |
| 12w-old vs. 24w-old | 0,0008 |  |  |  |  |
| 18w-old vs. 24w-old | 0,1446 |  |  |  |  |
| **Figure S1a** |  |  |  |  |  |
| **Two-way ANOVA** | **6w-old** | **24w-old** |  |  |  |
| 0 dpi vs. 2 dpi | 0,8305 | 0,9507 |  |  |  |
| 0 dpi vs. 7 dpi | 0,7156 | 0,1278 |  |  |  |
| 0 dpi vs. 21 dpi | 0,8803 | 0,414 |  |  |  |
| 2 dpi vs. 7 dpi | 0,9965 | 0,3154 |  |  |  |
| 2 dpi vs. 21 dpi | 0,9995 | 0,7286 |  |  |  |
| 7 dpi vs. 21 dpi | 0,9881 | 0,8868 |  |  |  |
|  | **0 dpi** | **2 dpi** | **7 dpi** | **21 dpi** |  |
| 6w-old vs. 24w-old | >0,9999 | 0,5487 | 0,0105 | 0,1142 |  |
| **Figure S1b** |  |  |  |  |  |
| **Two-way ANOVA** | **6w-old** | **24w-old** |  |  |  |
| 0 dpi vs. 2 dpi | 0,8692 | 0,495 |  |  |  |
| 0 dpi vs. 7 dpi | 0,9336 | 0,4488 |  |  |  |
| 0 dpi vs. 21 dpi | 0,991 | 0,2193 |  |  |  |
| 2 dpi vs. 7 dpi | 0,565 | 0,0444 |  |  |  |
| 2 dpi vs. 21 dpi | 0,7305 | 0,012 |  |  |  |
| 7 dpi vs. 21 dpi | 0,9905 | 0,9862 |  |  |  |
|  | **0 dpi** | **2 dpi** | **7 dpi** | **21 dpi** |  |
| 6w-old vs. 24w-old | >0,9999 | 0,9695 | 0,8174 | 0,3508 |  |
| **Figure S1c** |  |  |  |  |  |
| **Two-way ANOVA** | **6w-old** | **24w-old** |  |  |  |
| 0 dpi vs. 2 dpi | 0,9754 | 0,8482 |  |  |  |
| 0 dpi vs. 7 dpi | 0,9785 | 0,5512 |  |  |  |
| 0 dpi vs. 21 dpi | 0,1591 | 0,0778 |  |  |  |
| 2 dpi vs. 7 dpi | 0,8488 | 0,9362 |  |  |  |
| 2 dpi vs. 21 dpi | 0,0717 | 0,3262 |  |  |  |
| 7 dpi vs. 21 dpi | 0,3067 | 0,7342 |  |  |  |
|  | **0 dpi** | **2 dpi** | **7 dpi** | **21 dpi** |  |
| 6w-old vs. 24w-old | >0,9999 | 0,6528 | 0,8138 | 0,9931 |  |
| **Figure S1d** |  |  |  |  |  |
| **Two-way ANOVA** | **6w-old** | **24w-old** |  |  |  |
| 0 dpi vs. 2 dpi | 0,8781 | 0,2373 |  |  |  |
| 0 dpi vs. 7 dpi | 0,9853 | 0,3645 |  |  |  |
| 0 dpi vs. 21 dpi | 0,9813 | 0,0373 |  |  |  |
| 2 dpi vs. 7 dpi | 0,9819 | 0,999 |  |  |  |
| 2 dpi vs. 21 dpi | 0,9856 | 0,7602 |  |  |  |
| 7 dpi vs. 21 dpi | >0,9999 | 0,7217 |  |  |  |
|  | **0 dpi** | **2 dpi** | **7 dpi** | **21 dpi** |  |
| 6w-old vs. 24w-old | >0,9999 | 0,0733 | 0,2643 | 0,0225 |  |
| **Figure S1e** |  |  |  |  |  |
| **Two-way ANOVA** | **6w-old** | **24w-old** |  |  |  |
| 0 dpi vs. 2 dpi | 0,9038 | 0,7952 |  |  |  |
| 0 dpi vs. 7 dpi | 0,7028 | 0,2388 |  |  |  |
| 0 dpi vs. 21 dpi | 0,9851 | 0,2089 |  |  |  |
| 2 dpi vs. 7 dpi | 0,9773 | 0,739 |  |  |  |
| 2 dpi vs. 21 dpi | 0,9872 | 0,6925 |  |  |  |
| 7 dpi vs. 21 dpi | 0,8823 | 0,9998 |  |  |  |
|  | **0 dpi** | **2 dpi** | **7 dpi** | **21 dpi** |  |
| 6w-old vs. 24w-old | >0,9999 | 0,9988 | 0,8707 | 0,3664 |  |
| **Figure S1f** |  |  |  |  |  |
| **Two-way ANOVA** | **6w-old** | **24w-old** |  |  |  |
| 0 dpi vs. 2 dpi | 0,9966 | >0,9999 |  |  |  |
| 0 dpi vs. 7 dpi | 0,6522 | 0,4539 |  |  |  |
| 0 dpi vs. 21 dpi | 0,9807 | 0,1175 |  |  |  |
| 2 dpi vs. 7 dpi | 0,5763 | 0,4853 |  |  |  |
| 2 dpi vs. 21 dpi | 0,9416 | 0,1302 |  |  |  |
| 7 dpi vs. 21 dpi | 0,8595 | 0,8335 |  |  |  |
|  | **0 dpi** | **2 dpi** | **7 dpi** | **21 dpi** |  |
| 6w-old vs. 24w-old | >0,9999 | 0,9983 | 0,9957 | 0,226 |  |
| **Figure S1g** |  |  |  |  |  |
| **Two-way ANOVA** | **6w-old** | **24w-old** |  |  |  |
| 0 dpi vs. 2 dpi | 0,7638 | 0,6996 |  |  |  |
| 0 dpi vs. 7 dpi | 0,9462 | 0,9484 |  |  |  |
| 0 dpi vs. 21 dpi | 0,996 | 0,4016 |  |  |  |
| 2 dpi vs. 7 dpi | 0,439 | 0,9459 |  |  |  |
| 2 dpi vs. 21 dpi | 0,6332 | 0,9574 |  |  |  |
| 7 dpi vs. 21 dpi | 0,9878 | 0,7207 |  |  |  |
|  | **0 dpi** | **2 dpi** | **7 dpi** | **21 dpi** |  |
| 6w-old vs. 24w-old | >0,9999 | 0,1849 | >0,9999 | 0,5553 |  |
| **Figure S1h** |  |  |  |  |  |
| **Two-way ANOVA** | **6w-old** | **24w-old** |  |  |  |
| 0 dpi vs. 2 dpi | 0,5383 | 0,4843 |  |  |  |
| 0 dpi vs. 7 dpi | 0,9139 | 0,024 |  |  |  |
| 0 dpi vs. 21 dpi | 0,9979 | 0,0727 |  |  |  |
| 2 dpi vs. 7 dpi | 0,2322 | 0,0008 |  |  |  |
| 2 dpi vs. 21 dpi | 0,4417 | 0,0027 |  |  |  |
| 7 dpi vs. 21 dpi | 0,9642 | 0,9542 |  |  |  |
|  | **0 dpi** | **2 dpi** | **7 dpi** | **21 dpi** |  |
| 6w-old vs. 24w-old | >0,9999 | 0,0506 | 0,0041 | 0,0433 |  |

Abbreviations: dpi, days *post* injury; w, week; bioc, biocytin; DAPI, 4′,6-diamidino-2-phenylindole**.**

**Table S2: Primer sequences used for RT-qPCR analysis**

| **Gene** | **Primer sequence (5’ > 3’)** |
| --- | --- |
| *sdha* | F: gagagcattctaggacgtaaag |
|  | R: taggtgtgggtgagactatg |
| *hprt1* | F: ttgctggtgaagagaacac |
|  | R: cgttgtagtctagtgcgtatc |
| *gap43* | F: gagcagagtgaacagaagaag |
|  | R: actccaccactccatctac |
| *tuba1a* | F: atccatccattgtctgaacc |
|  | R: caacctcctcataatccttctc |
| *il-1β* | F: ccgacagcaagaaacgaa |
|  | R: gtggcaggacaggtataga |
| *tnf* | F: caggctcacaagaggttatt |
|  | R: ccagaggtcaatctgtcttatc |
| *il-6* | F: ggaggaatttcaagggaacata |
|  | R: cctcaggagaaccatgtaga |
| *il-8* | F: acaaatcctgaccacaagtag |
|  | R: atcgtattcaccatcatgtctc |

Abbreviations: RT-qPCR, real-time quantitative polymerase chain reaction; F, forward; R, reverse; *sdha*, *succinate dehydrogenase complex flavoprotein subunit a*; *hprt1*, *hypoxanthine phosphoribosyltransferase 1*; *gap43*, *growth-associated protein 43*; *tuba1a*, *tubulin alpha 1a chain; il, interleukin; tnf, tumor necrosis factor.*

**Table S3: Summary of the primary antibodies used for immunohistochemistry**

| **Antibody** | **Manufacturer** | **Catalog number** | **Host species** | **Monoclonal or polyclonal** | **Dilution** |
| --- | --- | --- | --- | --- | --- |
| Activated-caspase-3 | BioVision | 3015-100 | Rabbit | Polyclonal | 1:70 |
| Gfap | DAKO | Z0334 | Rabbit | Polyclonal | 1:200 |
| L-plastin | Genetex | GTX105789 | Rabbit | Polyclonal | 1:500 |
| Vimentin | Sigma-Aldrich | V5255 | Mouse | Monoclonal | 1:400 |
| Znp-1 | DSHB |  | Mouse | Monoclonal | 1:500 |
